# Orbital coupling of hetero-diatomic nickel-iron site for bifunctional electrocatalysis of CO_2_ reduction and oxygen evolution

**DOI:** 10.1038/s41467-021-24052-5

**Published:** 2021-07-02

**Authors:** Zhiping Zeng, Li Yong Gan, Hong Bin Yang, Xiaozhi Su, Jiajian Gao, Wei Liu, Hiroaki Matsumoto, Jun Gong, Junming Zhang, Weizhen Cai, Zheye Zhang, Yibo Yan, Bin Liu, Peng Chen

**Affiliations:** 1 State Key Laboratory of Optoelectronic Materials and Technologies, School of Materials Science and Engineering, Sun Yat-sen University, Guangzhou, China; 2 School of Chemical and Biomedical Engineering, Nanyang Technological University, Singapore, Singapore; 3 Institute for Structure and Function, Department of Physics, Chongqing University, Chongqing, China; 4 Institute for Materials Science and Devices, Suzhou University of Science and Technology, Suzhou, China; 5 Shanghai Synchrotron Radiation Facility, Shanghai Advanced Research Institute, CAS, Shanghai, China; 6 Dalian Institute of Chemical Physics, Chinese Academy of Sciences, Dalian, China; 7 Hitachi High-Technologies (Shanghai) Co. Ltd., Shanghai, People’s Republic of China; 8 Shaanxi Institute of Flexible Electronics, Northwestern Polytechnical University, Xi’an, China

**Keywords:** Electrocatalysis, Electrocatalysis, Nanoscale materials

## Abstract

While inheriting the exceptional merits of single atom catalysts, diatomic site catalysts (DASCs) utilize two adjacent atomic metal species for their complementary functionalities and synergistic actions. Herein, a DASC consisting of nickel-iron hetero-diatomic pairs anchored on nitrogen-doped graphene is synthesized. It exhibits extraordinary electrocatalytic activities and stability for both CO_2_ reduction reaction (CO_2_RR) and oxygen evolution reaction (OER). Furthermore, the rechargeable Zn-CO_2_ battery equipped with such bifunctional catalyst shows high Faradaic efficiency and outstanding rechargeability. The in-depth experimental and theoretical analyses reveal the orbital coupling between the catalytic iron center and the adjacent nickel atom, which leads to alteration in orbital energy level, unique electronic states, higher oxidation state of iron, and weakened binding strength to the reaction intermediates, thus boosted CO_2_RR and OER performance. This work provides critical insights to rational design, working mechanism, and application of hetero-DASCs.

## Abstract (web summary)

Diatomic site catalysts utilize two adjacent atomic metal species for their complementary functionalities and synergistic actions. Here, the authors report the orbital coupling of hetero-diatomic nickel-iron site boosts CO_2_ reduction reaction and oxygen evolution reaction.

## Introduction

Single-atom catalysts (SACs) have attracted tremendous interest for various reactions towards energy conversion and storage because of the ultimate atom utilization efficiency and high tunability of the electronic states through tailoring the coordination environment^1–9^. SACs are also recognized as ideal model systems to reveal the catalytic mechanisms at atomic level. For example, transition metal (TM) SACs have demonstrated good performance for CO_2_ reduction to CO (CO_2_RR), which in turn can be utilized to produce fuel or useful chemicals^10–19^. However, their practical use is limited by poor stability and low conversion efficiency. For instance, Ni-SAC on nitrogen-doped graphene for CO_2_RR offers a high intrinsic catalytic activity and Faradaic efficiency (FE) for CO production but suffers from the large onset potential due to the high energy barrier to form *COOH intermediate^19,20^. Fe-SAC exhibits a low onset potential for CO_2_RR. However, the strong binding of Fe site with reaction intermediates, such as CO, seriously compromises FE and stability^21^. Moreover, too strong binding strength of TM sites with electron-donating intermediates lowers the catalytic activity for oxygen evolution reaction (OER)^22,23^.

In contrast to SACs, diatomic site catalysts (DASCs) utilize two adjacent atomic metal species for their complementary functionalities and synergistic actions^24–28^. Particularly, the binding energy with the intermediates can be tailored by electronic interactions between two adjacent heteroatomic metal species. DASCs on nitrogenated carbon have been reported to perform well for CO_2_RR, oxygen reduction reaction (ORR), or hydrogen evolution reaction (HER)^24,29–32^. However, it remains as a great challenge to readily and rationally construct DASCs with high catalytic performance and multifunctionalities and understand their working mechanisms.

Here, we have developed a bifunctional DASC consisting of high-density Ni and Fe atoms anchored on N-doped graphene (NG) for both CO_2_RR and OER. NiFe-DASC displays extraordinary and stable electrocatalytic performance for CO_2_-to-CO conversion (50.4 mA cm^-2^ at an overpotential of 690 mV, FE of 94.5%) and O_2_ evolution (10 mA cm^-2^ at an overpotential of 310 mV), greatly outperforming Ni-SAC and Fe-SAC. The Zn-CO_2_ battery equipped with a NiFe-DASC cathode demonstrates high FE (90.6%) for CO_2_-to-CO reduction and outstanding rechargeable stability. The in-depth electronic structure analysis reveals that Fe of NiFe heteroatomic pair serves as the catalytic center and its orbital coupling with Ni leads to a higher oxidation state and weakened binding strength with the intermediates. This work provides critical insights to rational design, working mechanism, and application of hetero-DASCs.

## Results

### Hetero-diatomic site catalysts

**Fig. 1:**
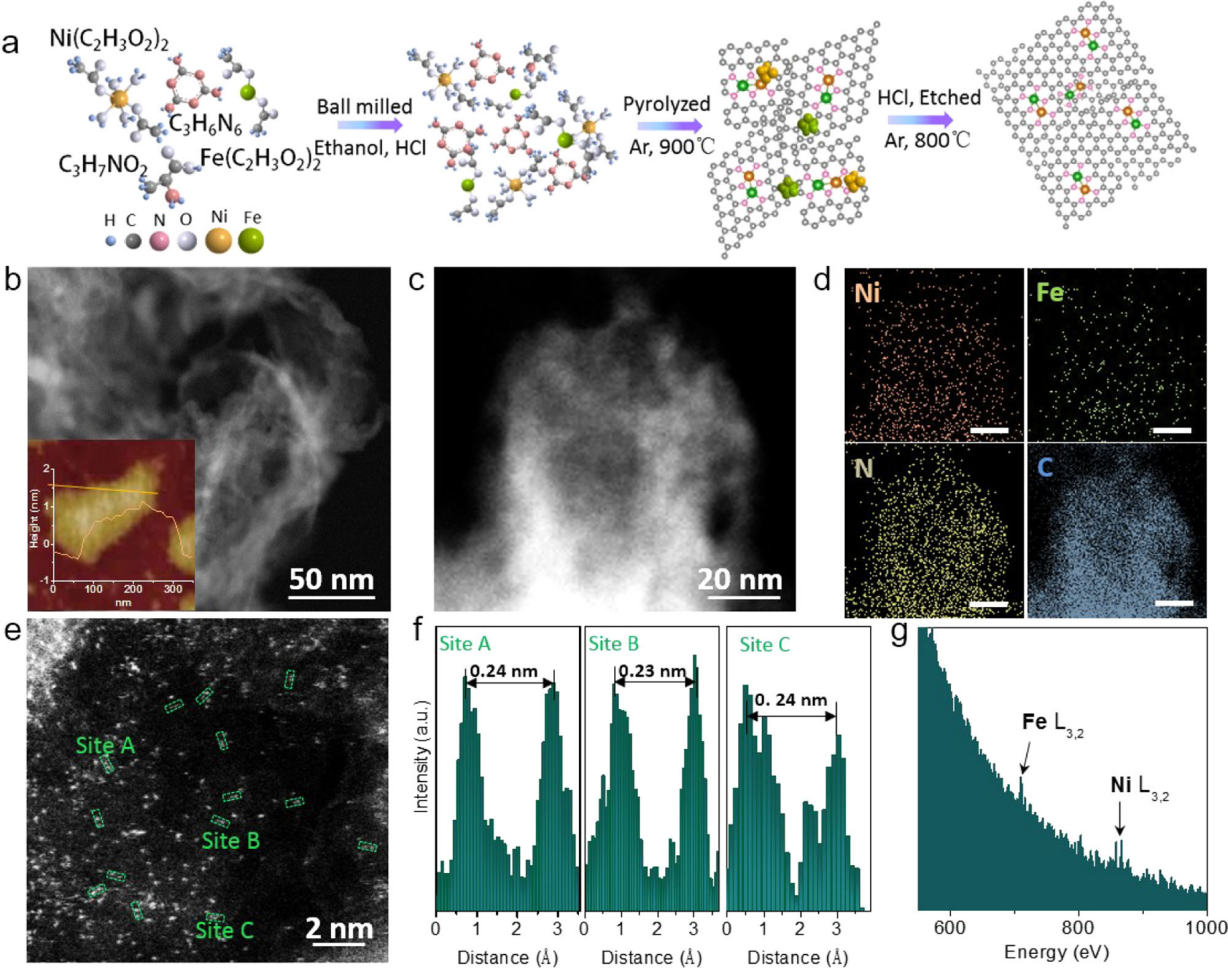
Synthesis and characterization of NiFe-DASC. **a** Schematic illustration of NiFe-DASC synthesis. **b** Dark-field scanning TEM and AFM (inset) images. **c** TEM image and **d** EDS elemental mapping of NiFe-DASC. **e** HAADF-STEM image of NiFe-DASC with some diatomic pairs being highlighted by green rectangles. **f** Intensity profiles of the three sites in **e**. **g** Electronic energy loss spectroscopy (EELS) of NiFe-DASC (706 eV for Fe-L_3,2_, 850 eV for Ni-L_3,2_ edges).

NiFe-DASC catalyst was synthesized by pyrolyzing l-alanine (amino acid), ferric (II) acetate, nickel (II) acetate tetrahydrate, and melamine together in argon atmosphere (Fig. 1a). Ni-SAC (or Fe-SAC) was prepared using the same method without adding Fe acetate (or Ni acetate). Before characterizations, all samples were grinded and then washed by 2 M HCl solution at 80 ^o^C for 24 h under stirring to remove metal particles. The X-ray diffraction (XRD) patterns of all three catalysts exhibit a strong peak at 28° and a weak peak at ~44° corresponding to (002) and (101) diffractions of graphitic carbon (Supplementary Fig. 2). No diffraction peaks related to metal or metal compounds can be observed. As revealed by dark-field scanning transmission electron microscopy (TEM) and scanning electron microscopy (SEM) (Fig. 1b and Supplementary Fig. 1), a porous architecture was formed by two-dimensional sheets. The sheet thickness of ~1 nm uncovered by atomic force microscopy (AFM) indicates the formation of single- or double-layered graphene (Fig. 1b, inset). Inductively coupled plasma-atomic emission spectroscopy (ICP-AES) reveals that the total metal contents in NiFe-DASC is 7.29 wt.% (4.05 wt.% of Ni and 3.24 wt.% of Fe), which approximately doubles that (3.3–3.5 wt.%) in Ni-SAC and Fe-SAC (Supplementary Table 1). The higher metal atom loading capacity of the diatomic catalysts implies that the two metal species may interact to stabilize each other. As shown in the dark-field scanning TEM and EDS elemental mapping of NiFe-DASC (Fig. 1c and Supplementary Fig. 3)_,_ both Ni and Fe atoms uniformly distribute on the entire NG support. As shown in Supplementary Fig. 4, their atomic distribution was also revealed by aberration-corrected high-angle annular dark-field scanning transmission electron microscopy (HAADF-STEM). As also seen, Ni and Fe atoms are uniformly dispersed on carbon support in Ni- and Fe-SAC, respectively. HAADF-STEM image of NiFe-DASC demonstrates a higher density of bright spots corresponding to Ni or Fe atoms (Fig. 1d). In addition, numerous atomic pairs are noticed with a typical distance of ~0.24 nm (Fig. 1e), implying the possible formation of atomic pairs via metal–metal (Ni–Fe) bonds. Electron energy loss spectroscopy (EELS) obtained in one acquisition further confirms that the bright spots correspond to Ni and Fe atoms (Fig. 1g and Supplementary Fig. 5). Raman spectrum of NiFe-DASC contains a characteristic G band at 1576 cm^-1^ and a D band at 1362 cm^-1^, resulting from sp^2^-hybridized carbon atoms and carbon lattice defects, respectively (Supplementary Fig. 6). NiFe-DASC displays a larger specific surface area (433.2 m^2^ g^-1^) than that of Ni-SAC (340.9 m^2 ^g^-1^) and Fe-SAC (302.7 m^2 ^g^-1^) (Supplementary Fig. 7).

**Fig. 2:**
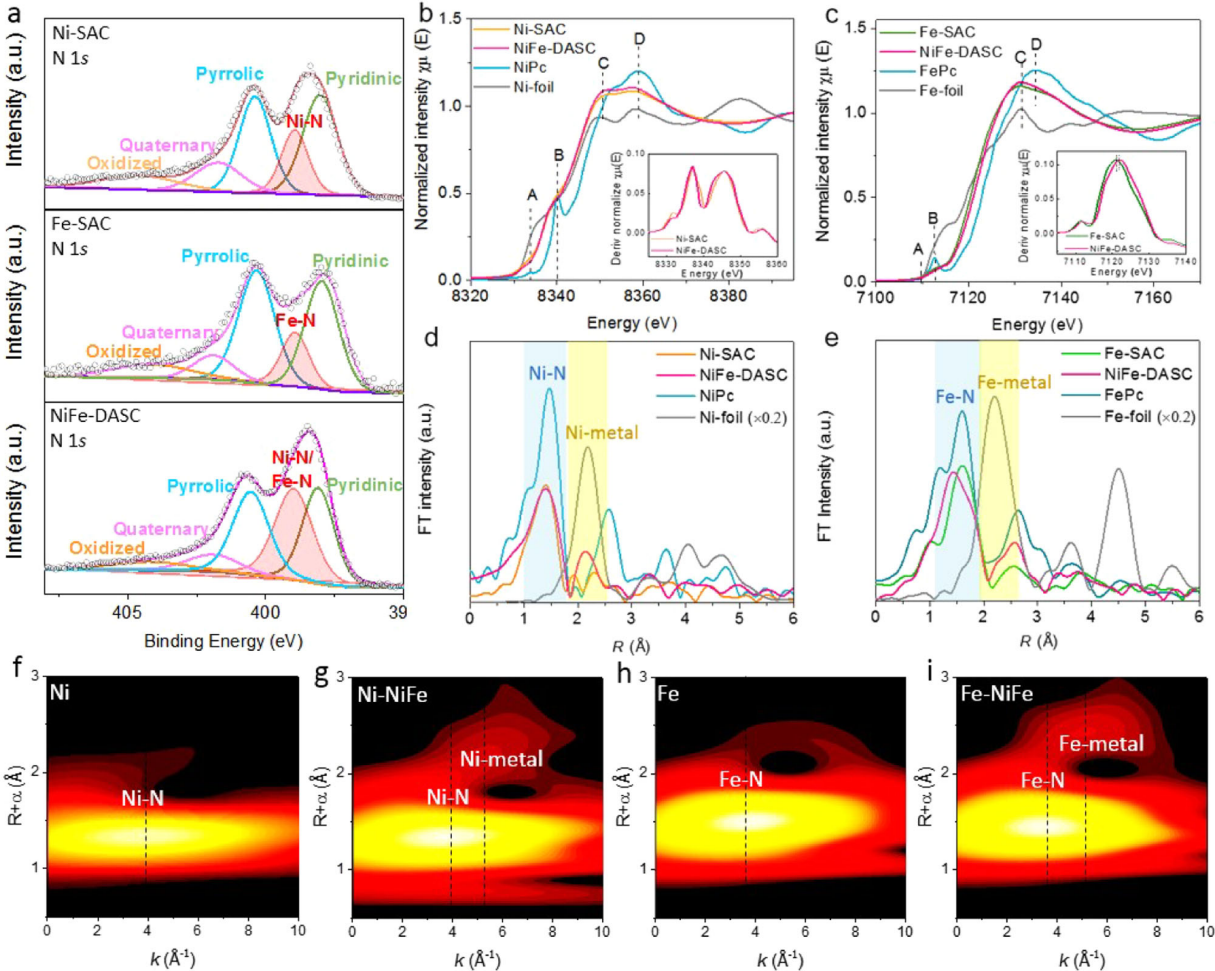
XPS and XAS characterizations of the catalysts. **a** High-resolution XPS spectra of N 1*s*. **b** Ni K-edge XANES spectra. **c** Fe K-edge XANES spectra. Peak A, B, C, D in **b** and **c** represent 1*s* → *3d* transition, 1*s* → 4*p*_*z*_ transition, 1*s* → 4*p*_*x,y*_ transition and multiple scattering processes, respectively. Insets of **b** and **c** are the first derivatives of Ni and Fe K-edge of XANES spectra, respectively. Fourier transformation (FT)-EXAFS spectra of **d** Ni-SAC and NiFe-DASC, **e** Fe-SAC and NiFe-DASC. Wavelet transform (WT)-EXAFS of k^2^-weighted k-space spectra of Ni for **f** Ni-SAC and **g** NiFe-DSAC, of Fe for **h** Fe-SAC and **i** NiFe-DSAC.

Elemental chemical states were revealed by X-ray photoelectron spectroscopy (XPS). In the high-resolution XPS spectra of NiFe-DASC, Ni signal (854.7 eV) was similar to that from Ni-SAC, whereas Fe peak (710.7 eV) is up-shifted as compared to that of Fe-SAC (710.2 eV), indicating the higher oxidation state of Fe in the DASC (Supplementary Fig. 8). The N 1*s* spectra of the three catalysts show the existence of pyridinic (~398.1 eV), Ni-N/Fe-N (~399.0 eV), pyrrolic (~400.7 eV), quaternary (~402.0 eV) and oxidized (~404.2 eV) N species (Fig. 2a). The contents of metal-N species are 20.2%, 15.8% and 36.7% in all N species for Ni-SAC, Fe-SAC, and NiFe-DASC, respectively, being consistent with the observation that NiFe-DASC has a higher metal content and suggesting that the transition metal atoms are anchored on graphene via metal-N coordination. In contrast, no obvious peak of metal-N species can be observed in N-doped graphene (Supplementary Fig. 9). X-ray absorption near-edge structure (XANES) and extended X-ray absorption fine structure (EXAFS) measurements were carried out to further investigate the physicochemical characteristics of Ni and Fe atoms in NiFe-DASC. As shown in Fig. 2b, the rising edges of Ni K-edge XANES spectra for Ni-SAC and NiFe-DASC are nearly identical, which is also evidenced by the identical first derivatives of the spectra (inset of Fig. 2b). Moreover, these spectra locate between the Ni K-edge spectra from nickel (II) phthalocyanine (NiPc) and Ni-foil, suggesting that the oxidation state of Ni atoms in Ni-SAC and NiFe-DASC is +1 with a 3d^9^, *S* = 1/2 electronic state. The intensity of pre-edge peak A (~8333.5 eV) for NiFe-DASC is slightly lower than that of Ni-SAC, implying a small distortion of D_4h_ symmetry for Ni atoms in NiFe-DASC. As depicted in Fig. 2c, the rising edge of Fe K-edge spectra for NiFe-DASC shifts ~+0.6 eV, as compared to that of Fe-SAC, indicating a higher oxidation state of Fe atom in NiFe-DASC, agreeing with the finding from XPS. Figure 2d,e display the magnitude of Fourier transform of k^2^-weighted EXAFS spectra (uncorrected for phase shifts) and the corresponding k^2^-weighted EXAFS spectra are shown in Supplementary Fig. 10. The predominant peaks for Ni-SAC and NiFe-DASC, which originate from the scattering of first shell Ni–N path, are almost the same in position (at ~1.45 Å) and magnitude, indicating nearly identical coordination environment for Ni atoms in these two samples. Notably, the peak appears at ~2.2 Å in NiFe-DASC (just as in Ni foil) suggests the presence of Ni-metal diatomic configuration. On the other hand, Fe-metal bond evidenced by EXAFS spectrum of Fe K-edge for NiFe-DASC suggests the formation of Fe-metal diatomic configuration. Taken together, Ni–Fe diatomic configuration is formed. Compared to Fe-SAC, the first shell scattering (Fe-N) for NiFe-DASC displays asymmetry and slightly decreased magnitude, indicating that the chemical state of Fe is altered by the coupling Ni atom. In contrast, the difference of Ni-N scattering between Ni-SAC and NiFe-DASC is small. Wavelet transform (WT)-EXAFS was also conducted to identify the metal-N and metal–metal paths (Fig. 2f–i). Both Ni-SAC and NiFe-DASC show intensity maxima at 3.9 Å^-1^ for Ni-N path in Ni K-edge reference, while Fe-SAC and NiFe-DASC display the intensity maxima at 3.8 Å^-1^ for Fe-N path in Fe K-edge reference. For NiFe-DASC, both WT signals derived from Ni-metal bond and Fe-metal bond are located at ~5.2 Å^-1^, suggesting the formation of Ni–Fe bond. Although metal–metal (Ni–Fe) bond length in NiFe-DASC is similar to Ni–Ni bond length in Ni Foil (Fig. 2d) and Fe–Fe bond length in Fe foil (Fig. 2e), WT signals from Ni–Ni bond and Fe–Fe bond are significantly different (~7.0 Å^-1^, Supplementary Fig. 11), indicating distinctive bonding conditions in DASC. WT signals at Ni and Fe K-edge are consistent with the FT-EXAFS results, confirming existence of metal-N coordination and metal–metal bonds in NiFe-DASC.

### Electrocatalysis of CO_2_RR and OER

**Fig. 3:**
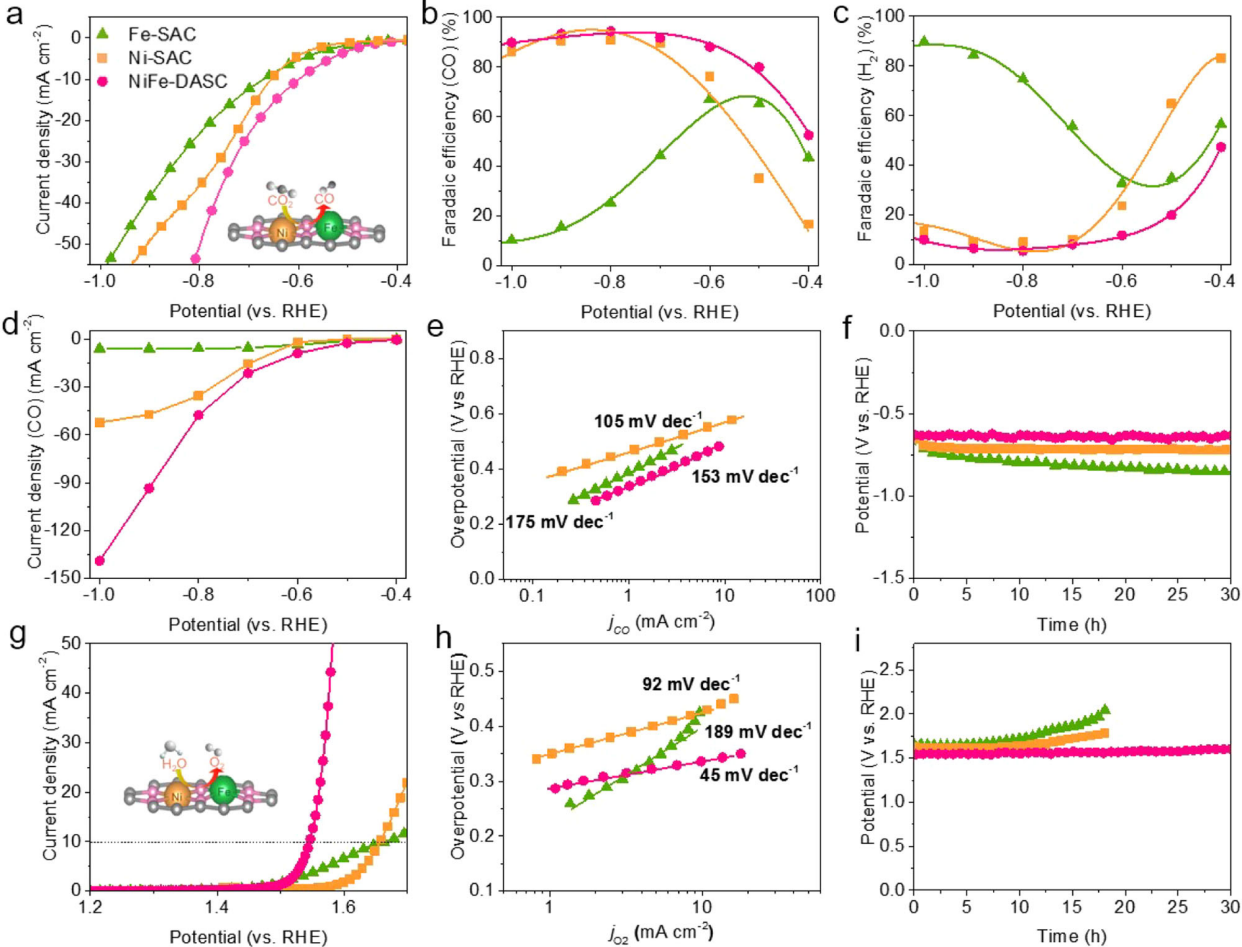
Bifunctional electrocatalysis of CO_2_ reduction and oxygen evolution. **a** Linear sweep voltammograms (LSVs) of Fe-SAC, Ni-SAC, and NiFe-DASC acquired on a rotating disc electrode at a rotation speed of 1600 r.p.m. and a scan rate of 5 mV s^-1^. Catalyst loading is 0.1 mg cm^-2^ and electrolyte is CO_2_-saturated 0.5 M KHCO_3_ solution. Faradaic efficiencies of **b** CO and **c** H_2_ generation at various potentials. **d** Partial current density of CO (*j*_CO_) in potentiostatic electrolysis. **e** Tafel plots for CO_2_RR. **f** Potential-time response for CO_2_RR at a current density of 10 mA cm^-2^. **g** LSV curves, and **h** Tafel plots for OER in O_2_-saturated 1.0 M KOH at a rotation speed of 1600 r.p.m and a scan rate of 1 mV s^-1^. **i** Potential-time response for OER at a current density of 10 mA cm^-2^.

The electrochemical activities of the atomic catalysts for CO_2_RR were evaluated by the linear sweep voltammetry (LSV) (Fig. 3a) and cyclic voltammograms (CV) (Supplementary Fig. 12) in a three-electrode electrolysis cell. As compared with Fe-SAC and Ni-SAC, NiFe-DASC possesses a larger electrochemically active surface area (ECSA), accounting for its higher catalytic activity (Supplementary Fig. 13). Below −0.40 V vs. RHE, NiFe-DASC produced higher current densities as compared with Ni-SAC and Fe-SAC. As shown in Supplementary Fig. 14, Fe-SAC exhibits a much lower onset potential than Ni-SAC (260 vs. 350 mV vs. RHE) and interestingly it is slightly inferior to NiFe-DASC (210 mV vs. RHE). On the other hand, Ni-SAC offers a higher FE for CO production in potential range from −0.6 to -1.0 V vs. RHE as compared to Fe-SAC (Fig. 3b). Also, interestingly it is slightly inferior to NiFe-DASC, which offers *a* > 80% FE at the same potential range with the maximum of 94.5% at −0.8 V vs. RHE. The high selectivity of NiFe-DASC is testified by the low FE and small partial current density (j_H2_) for HER (Fig. 3c and Supplementary Fig. 15). Noteworthy, NiFe-DASC unites the low overpotential of Fe-SAC and high selectivity of Ni-SAC. The CO partial current density (*j*_CO_) of NiFe-DASC (e.g., 98 mA cm^-2^ at an overpotential of 0.8 V) is much larger than that of Ni-SAC (48 mA cm^-2^) and Fe-SAC (6.7 mA cm^-2^) (Fig. 3d), suggesting the fast kinetics of NiFe-DASC. The Tafel slope of NiFe-DASC (153 mV dec^-1^) is lower than that of Fe-SAC (175 mV dec^-1^), indicating the improved CO_2_RR kinetics after incorporation of Ni atoms (Fig. 3e). The calculated turnover frequency (TOF) of NiFe-DASC is 15,055 h^-1^ at an overpotential of 0.68 V, which is larger than those of Ni-SAC (10179 h^-1^) and Fe-SAC (1731 h^-1^). Moreover, CO_2_RR by NiFe-DASC is stable, retaining >90% of the initial potential level at 10 mA cm^-2^ after 30 hours of continuous operation (Fig. 3f), whereas the potential for Fe-SAC decreases largely presumably due to the strong CO binding with the Fe site and resultant poisoning. This problem is alleviated in NiFe-DASC likely because CO binding is weakened by neighboring Ni atom. For comparison, the electrochemical activities of physically mixed Ni- and Fe-SAC (m-Ni,Fe-SAC) were also evaluated (Supplementary Fig. 16). m-Ni/Fe-SAC exhibited lower current densities and a lower Faradaic efficiency for CO production as compared with NiFe-DASC, further testifying the advantage of hetero-diatomic site in NiFe-DASC. Based on the above analyses, NiFe-DASC demonstrates outstanding electrocatalytic activity, selectivity and stability towards CO_2_RR, which is superior or comparable to other state-of-the-art catalysts (Supplementary Table 2).

The OER performance of NiFe-DASC was evaluated in 1.0 M KOH electrolyte on a rotating disk electrode (RDE) (Fig. 3g). NiFe-DASC achieves a current density of 10 mA cm^-2^ at a low potential of +1.54 V vs. RHE, outperforming Ni-SAC ( + 1.65 V vs. RHE) and Fe-SAC ( + 1.67 V vs. RHE), as well as other state-of-the-art catalysts (Supplementary Table 3). Moreover, NiFe-DASC has the lowest Tafel slope (45 mV dec^-1^) as compared with Ni-SAC (92 mV dec^-1^) and Fe-SAC (189 mV dec^-1^) (Fig. 3h), suggesting that it offers the fastest reaction kinetics. After 50th cyclic voltammetry (CV), an obvious potential increase for Ni-SAC (21 mV) and Fe-SAC (52 mV) at the current density of 5 mA cm^-2^ were recorded (Supplementary Fig. 17). Such catalyst instability could be attributable to oxidation of single atoms by the strongly adsorbed oxygen-containing intermediates^25^. In contrast, NiFe-DASC essentially retained its initial OER activity, indicating its better durability in alkaline media. Moreover, negligible performance degradation of NiFe-DASC was observed at 10 mA cm^-2^ over 30 h of continuous electrolysis, superior to Ni-SAC and Fe-SAC (Fig. 3i). In sum, NiFe-DASC gives high OER activity with a low overpotential and excellent long-term stability.

### Rechargeable zinc-CO_2_ battery

**Fig. 4:**
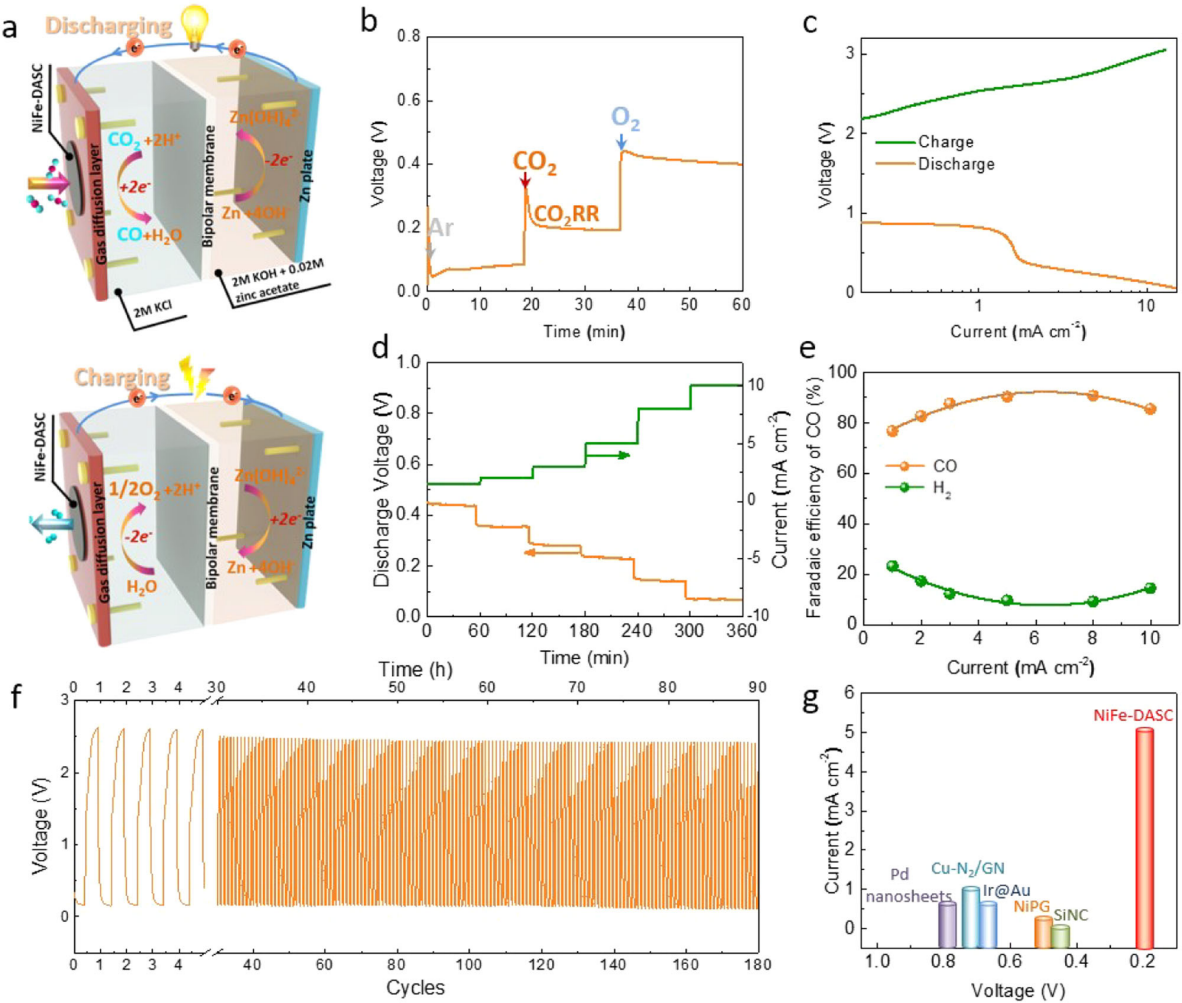
The rechargeable Zn-CO_2_ battery cell. **a** Schematic illustration of the designed battery cell. **b** Chronopotentiometric curve of NiFe-DASC at a current density of 5 mA cm^-2^, with gas supply of Ar for the first 20 min, subsequently CO_2_ between 20-40 min, and finally O_2_. **c** Discharge and charge polarization curves at a scanning rate of 10 mV s^-1^. **d** Discharge voltages at different currents and **e** Faradaic efficiencies of CO and H_2_ generation upon battery discharging. **f** Galvanostatic discharge-charge cycling curves at the discharge current density of 5 mA cm^-2^ and charge current density of 2 mA cm^-2^ for 180 cycles. **g** Comparison of discharge performance of Zn-CO_2_ batteries using different catalysts (also see Supplementary Table 4).

Rechargeable metal-CO_2_ batteries, which integrate the working principles of CO_2_RR and metal-air batteries, have recently been proposed as a new sustainable power technology with additional benefit of promoting carbon recycling^33–37^. With outstanding catalytic activities of CO_2_RR and OER, NiFe-DASC is desirable as the electrode catalyst in metal-CO_2_ batteries. The rechargeable Zn-CO_2_ battery cell was assembled with carbon paper loaded with the bifunctional NiFe-DASC catalyst as the cathode and Zn plate as the anode (Fig. 4a and Supplementary Fig. 18). As shown in Fig. 4b, at the discharge current density of 5 mA cm^-2^, the discharge potentials of HER, CO_2_RR, and ORR, stabilize at 0.08, 0.21, and 0.42 V, respectively. The discharge (charge) voltages reach in a range of 0.89–0.12 V (2.16–3.01 V) at a current density of 0.1–10 mA cm^-2^, indicating the practicality of our rechargeable Zn-CO_2_ battery (Fig. 4c). It is noted that the catalytic activity and voltage efficiency during electrochemical conversion of CO_2_ in battery device is rather different to that in the Zn-O_2_ battery (Supplementary Fig. 19). The discharge polarization curves clearly exhibit the discharge voltage during ORR is higher than that in CO_2_RR. At the constant discharge current density of 1.5 mA cm^-2^, the discharge voltage is 0.43 V and gradually decreases with increasing current (Fig. 4d). As demonstrated in Fig. 4e, FE of CO production reaches 90.6% at 5 mA cm^-2^ and still remains at 85% when the discharge current density increases to 10 mA cm^-2^, suggesting that CO_2_-to-CO conversion dominates in a wide current range (2–10 mA cm^-2^). The sum of the FEs for CO and H_2_ generation is ~100%, excluding the possibility of other products during discharge. The galvanostatic discharge-charge cycling at the discharge current density of 5 mA cm^-2^ and charge current density of 2 mA cm^-2^ gives stable voltage output during 180 cycles, testifying the outstanding rechargeability (Fig. 4f). The galvanostatic discharge-charge cycling test at the current density of 1 mA cm^-2^ was also conducted, which showed the stable voltage output over 200 cycles (Supplementary Fig. 20). The battery equipped with NiFe-DASC cathode offers the largest discharge voltage and the lowest charge voltage as compared to the batteries with Ni-SAC or Fe-SAC cathode (Supplementary Fig. 21). Our Zn-CO_2_ battery can be used under large discharge current densities (over 10 mA cm^-2^, Supplementary Fig. 22), while other reported devices can only operate well under a low current density (~0.56 mA cm^-2^)^35,38–40^. At a low current density (e.g., 1 mA cm^-2^), the discharge voltage of our battery is 0.89 V, which is higher than the other devices based on different catalysts (e.g., Cu-N_2_/GN). Clearly, Zn-CO_2_ battery based on NiFe-DASC significantly outperforms others (Fig. 4g and Supplementary Table 4). It can achieve a maximum power density of 1.36 mW cm^-2^ at 8.5 mA cm^-2^ (Supplementary Fig. 23).

## Discussion

### Electronic structure of hetero-DASC

**Fig. 5:**
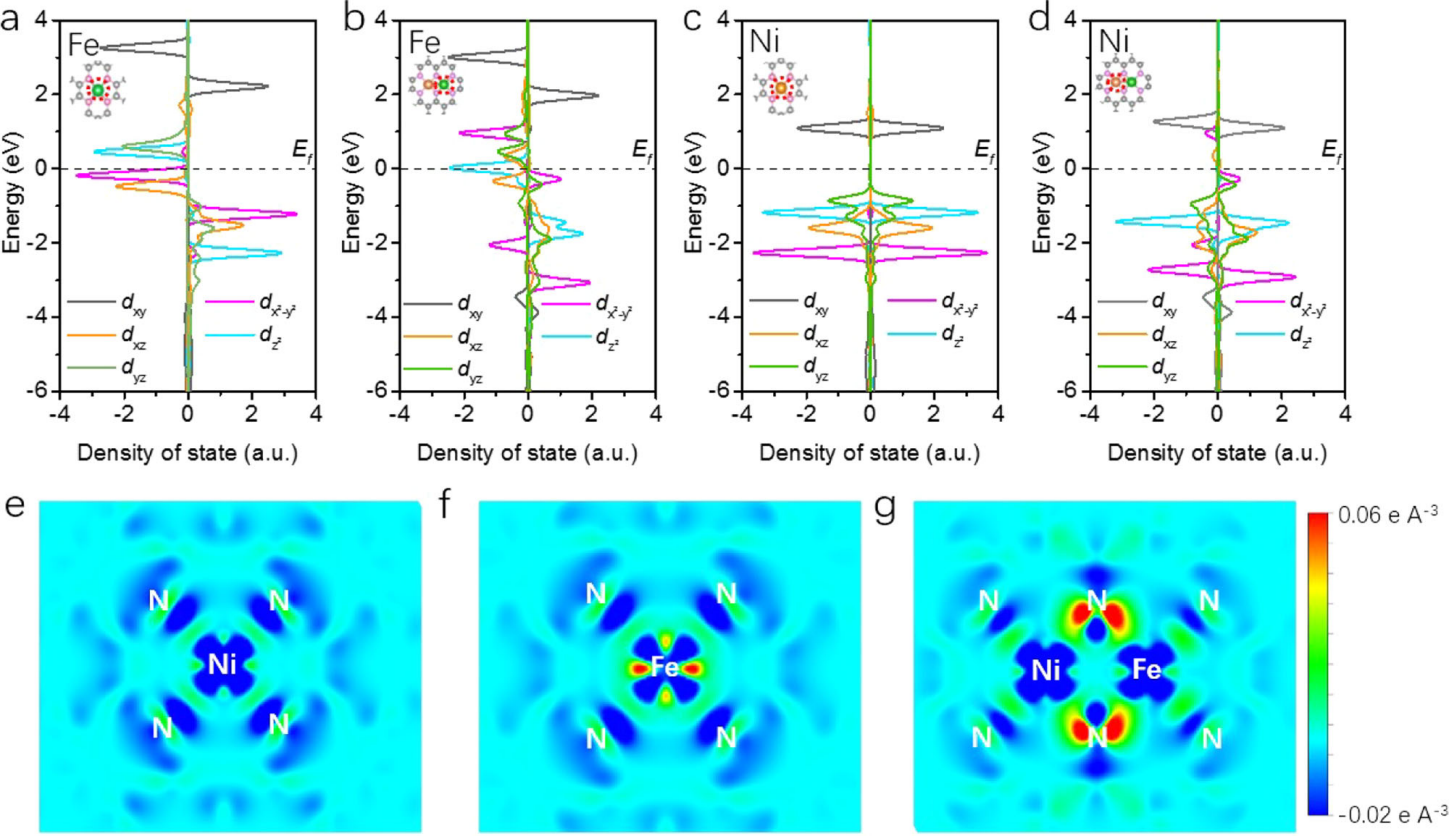
Electronic structure of single-atomic and hetero-diatomic catalyst. Density of states of Fe 3*d* for **a** Fe-SAC and **b** NiFe-DASC, of Ni 3*d* for **c** Ni-SAC and **d** NiFe-DASC. The differential charge density maps of **e** Ni-SAC, **f** Fe-SAC, and **g** NiFe-DASC. From aquamarine to orange indicates the transition from electron depletion to accumulation.

Density of states (DOS) of 3d orbitals of Fe and Ni centers in the SACs and DASC were derived by density functional theory (DFT) calculations as shown in Fig. 5a–d. As compared to Fe-SAC, 3*d* states of Fe in NiFe-DASC is less localized, especially for *d*_xz_ and *d*_yz_ orbitals. Moreover, *d*_z_^2^ orbital state crosses the Fermi level after incorporation of Ni atom. Such redistribution of Fe 3*d* states for NiFe-DASC could be understood by analyzing the orbital interactions between Ni, Fe, and N atoms (Supplementary Fig. 24). In NiFe-DASC, the 3*d*_x_^2^_-y_^2^ and 3*d*_z_^2^ orbitals of Fe and Ni resonate at ~1.5 and ~−1.8 eV, indicating a strong *d-d* orbital coupling between the heteroatoms. The strong interactions between Ni and Fe atoms in diatomic NiFe catalyst are also evidenced by the obvious energy level change of *d*_z_^2^, *d*_x_^2^_-y_^2^, *d*_xz_, *d*_xy_-*p*_z_*p*_y_, and *d*_yz_-*p*_x_ orbitals (Supplementary Fig. 25). The *d-d* (especially, *d*_z_^2^, *d*_x_^2^_-y_^2^, and *d*_xz_,) interactions between Ni and Fe cause decease of orbital energy levels and delocalization of electrons, which are beneficial to *CO desorption. Further analysis indicates that the difference of Ni 3*d* orbitals between Ni-SAC and NiFe-DASC is small (Fig. 5c, d), and DOS of *p*_z_ orbital for the coordinating N atom exhibits a significant increase (Supplementary Fig. 26). The alterations in DOS of Ni, Fe and N atoms in NiFe-DASC indicate electron transfer from Fe to Ni and N, mostly to *p*_z_ orbital of N atom via orbital coupling. This leads to a higher oxidation state of Fe center, which is in good agreement with the XAS and XPS results. In addition, the differential charge density maps in Fig. 5e–g show that, as compared to Fe-SAC, more electrons are delocalized around coordinating N between Ni and Fe, and the electrons from Fe are partially transferred to coordinating N. This results in a larger value of Bader charge (Supplementary Table 5) and a higher oxidation state of Fe center in NiFe-DASC, which consequently promote CO_2_ activation and CO desorption^10^. Moreover, the electronic state in hetero-diatomic catalyst was further investigated by ultraviolet photoelectron spectroscopy (UPS) (Supplementary Fig. 27). The binding energy of the second electron cutoff edge for NiFe-DASC is higher than that for Fe-SAC, indicating a decrease of work function that consequently promotes electron transfer to CO_2_ to form *COOH. This result suggests that a lower Gibbs free energy is required for the formation of *COOH. NiFe-DASC exhibits high-density states in the region of 0 to 5 eV as compared with Ni- and Fe-SAC. This is consistent with the DFT calculation, which shows that DOS for the active center in NiFe-DASC exhibits a larger electron occupancy near the Fermi level (Supplementary Fig. 28).

### Orbital interactions between adsorbed CO and Fe center

**Fig. 6:**
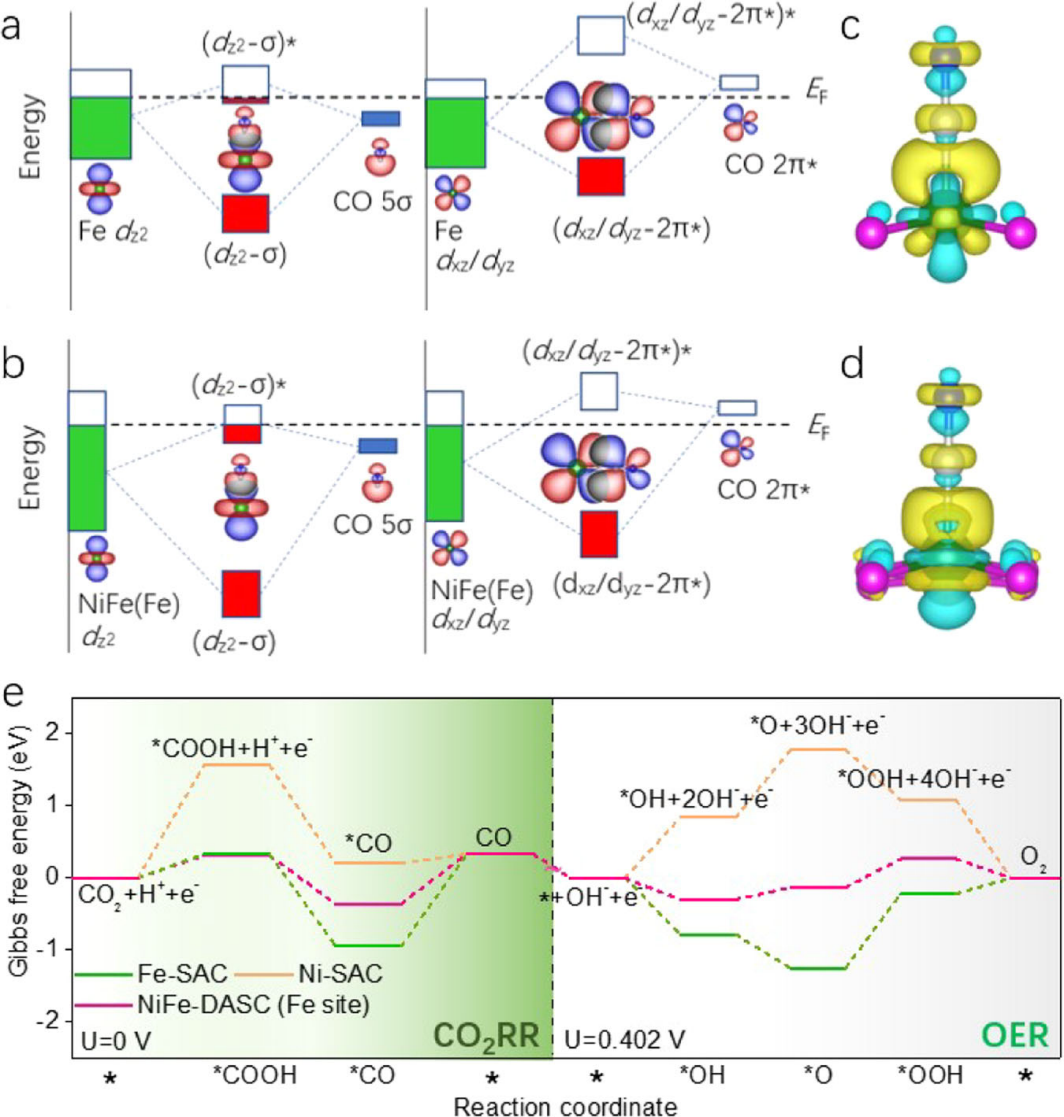
Orbital interactions between adsorbed CO and Fe center and free energy diagrams. Schematic illustration of orbital interactions between adsorbed CO (5σ and 2π*) and 3*d* orbital (*d*_z_^2^, *d*_xz_/*d*_yz_) of Fe site in **a** Fe-SAC and **b** NiFe-DASC. Differential charge density on **c** Fe-SAC-CO and **d** NiFe-DASC-CO. The cyan and yellow indicate electron depletion and accumulation, respectively. Green, brown, gray, blue, and pink balls present Fe, Ni, C, O, and N atoms. The value of isosurface is 0.001 e bohr^−3^. **e** Calculated free energy diagrams for CO_2_RR and OER processes.

Given that the rate limited step of CO_2_RR for Fe-SAC concerns the desorption of CO intermediate on Fe site^22^, the orbital interactions between Fe center and CO intermediate were further analyzed. Supplementary Fig. 29 shows the calculated DOS of adsorbed CO (5σ and 2π*) and 3d orbital (*d*_z_^2^, *d*_*x*z_/*d*_y*z*_) of Fe site in Fe-SAC and NiFe-DASC. The DOSs of Fe *d*_z_^2^ and CO 5σ (Fe *d*_xz_/*d*_yz_ and CO 2π*) exhibit substantial overlaps. Such interactions are strong enough to split into bonding and antibonding orbitals (Fig. 6a, b). Notably, the d band center of Fe in NiFe-DASC (−2.98 eV) locates lower than that in Fe-SAC (−2.48 eV), which leads to decrease energy levels of bonding and antibonding states of both *d*_z_^2^-5σ and *d*_xz_/*d*_yz_-2π* in NiFe-DASC. As a result, the corresponding antibonding state would show a larger electron occupancy. Moreover, change of charge redistribution upon CO adsorption is illustrated in Fig. 6c, d. Evidently, charge transfer is lesser in NiFe-DASC than Fe-SAC. It is noted that the interactions between Fe and *COOH on Fe-SAC and NiFe-DASC are almost identical (Supplementary Fig. S30), suggesting the similar binding strength of *COOH on the two catalysts. Taken together, our findings clearly demonstrate that incorporation of Ni leads to a weaker binding strength of *CO without altering the binding strength of *COOH on Fe center, whereby boosting the catalytic CO_2_RR activity. This may also be beneficial for OER process because of the likely weak binding of Fe center with electron-donating OER intermediates (scaling relationship)^23^.

Potential limiting steps on Fe center. The free energy diagrams for CO_2_RR and OER processes of Fe-SAC, Ni-SAC, and NiFe-DASC were investigated as shown in Fig. 6e. The potential limiting steps (PLSs) for CO_2_RR on Ni-SAC and Fe-SAC are CO_2_ → *COOH (1.56 eV) and *CO → CO (1.28 eV), because of large formation energy of *COOH on Ni-SAC and the high desorption energy of *CO on Fe-SAC. In comparison, Gibbs free energy (∆*G*) for the formation of *COOH intermediate on Fe site of NiFe-DASC is only 0.31 eV. The consequently enhanced *COOH formation is consistent with the experimental observation that the onset potential of CO_2_RR enabled by NiFe-DASC is lower (Supplementary Fig. 14). Moreover, ∆*G* of CO desorption on Fe site of NiFe-DASC is significantly lower than that on Fe-SAC (0.69 vs. 1.28 eV), which promotes CO production. The difference in ∆*G* of intermediate adsorption on Fe site for Fe-SAC and NiFe-DASC can be ascribed to the different adsorption behaviors of *COOH and *CO. Specifically, C=O bond of *COOH tilts to the bond of hetero-diatomic site, while *CO adsorption prefers end-on adsorption. The O atom in C=O of *COOH exhibits an interaction with hetero-diatomic center and thus contributes to the binding strength, which reduces ∆*G* of *COOH adsorption over the hetero-diatomic site. Furthermore, as shown in Supplementary Fig. 31, PLS of both Ni-Ni and Fe-Fe DASCs is CO_2_ → *COOH, and the corresponding ∆*G* (1.55 and 0.92 eV) are larger than that on NiFe-DASC, highlighting that the hetero-atom coupling is beneficial. As the formation of intermediates (*COOH and *CO) and desorption of CO occur on Fe site, the catalytic site in NiFe-DASC is Fe whose activities are largely enhanced by the coupling Ni (Supplementary Fig. 32).

Thermodynamic limiting potential (*U*_L_) is the highest potential in free energy diagram. Considering that HER is the dominant competitive reaction to CO_2_RR, the difference between *U*_L_ for CO_2_RR (*U*_L_(CO_2_RR)) and HER (*U*_L_(HER) (Fig. 6e and Supplementary Fig. 33) reflects the catalyst selectivity^24,41^. As expected, Δ*U*_L_ = *U*_L_(CO_2_RR)-*U*_L_(HER) for NiFe-DASC is more positive than that for Fe-SAC and Fe-Fe DASC (Supplementary Fig. 34 and Table 6), suggesting that NiFe-DASC is highly selective towards CO_2_RR. Although Δ*U*_L_ of Ni-SAC or Ni-Ni DASC is even more positive than that of NiFe-DASC, their CO_2_RR activity is hindered by the high energy barrier for the formation of *COOH.

For OER, PLSs on Ni-SAC (Ni-Ni DASC) and Fe-SAC (Fe-Fe DASC) are *OH → *O and *O → *OOH with ∆*G* of 0.94 eV (1.21 eV) and 1.03 eV (0.43 eV), respectively. In comparison, ∆*G* of PLS (*O → *OOH) over Fe site decreases to 0.39 eV on NiFe-DASC, meaning a higher catalytic activity towards OER. The formation energies of all reaction intermediates in OER on Fe site are much lower than those on Ni site in NiFe-DASC (Supplementary Table 8), suggesting that the Fe site rather than the neighboring Ni site is the active catalytic center for every catalytic step (Supplementary Fig. 35). Furthermore, the volcano plot for OER (Supplementary Fig. 36) is established using the overpotential (*ŋ*_OER_) vs. the well-accepted descriptor (the difference of ∆*G* between the formation of *O and *OH)^25^. NiFe-DASC appears at the top of the plot, indicating its outstanding OER performance.

In summary, we have demonstrated a diatomic NiFe catalyst supported by nitrogen-doped graphene with extraordinary and stable electrocatalytic performance towards CO_2_RR (94.5% FE of CO and 50.4 mA cm^-2^ at an overpotential of 690 mV) and OER (10 mA cm^-2^ at an overpotential of 310 mV). The rechargeable Zn-CO_2_ battery equipped with a NiFe-DASC cathode exhibits a high Faradaic efficiency (90% at 5 mA cm^-2^) as well as outstanding rechargeability, and can be used practically under large discharge current densities (over 10 mA cm^-2^). NiFe-DASC unites the merits of both Fe-SAC and Ni-SAC (specifically, low overpotential of the former and high selectivity of the latter) and is more stable than SACs. The state-of-the-art experimental characterizations and in-depth theoretical analyses attribute such superior performance to the orbital coupling (mainly 3*d*_x_^2^_-y_^2^ and 3*d*_z_^2^) between the catalytic Fe center and the adjacent Ni atom, which leads to higher oxidation state of Fe (due to the electron transfer from Fe to Ni and N) and weakened binding strength with the intermediates (mainly due to the lower energy levels of bonding and antibonding states of both *d*_z_^2^-5σ and *d*_xz_/*d*_yz_-2π*). This work provides critical insights to rational design, working mechanism, and application of hetero-DASCs.

## Methods

### Synthesis of transition metal DASCs

In all, 2 g of melamine, 2 g of l-alanine, and 50 mg of transition metal acetates (mole ratio of ferric(II) acetate/nickel(II) acetate = 1:1) were homogeneously mixed by ball milling for 30 min. Subsequently, 15 mL of ethanol mixed with 3 mL of hydrochloric acid was added and the slurry was then put in a mortar. It was milled in a fume hood until the ethanol was evaporated. The resultant solid was dried in an oven at 60 °C overnight and ball milled again for 30 min. The obtained powder was pyrolyzed under flowing Ar atmosphere in a tube furnace with the following ramping program: heated from room temperature to 600 °C at a rate of 2.5 °C min^-1^, held at 600 °C for 2 h, ramped to 900 °C at 5 °C min^-1^ and held for 1.5 h, finally naturally cooled down to room temperature. The obtained black solid materials were grinded and then washed by 2 M HCl solution at 80 °C for 24 h under stirring to remove metal particles. The materials were dried and then annealed again in argon at 800 °C for 1 h at a ramping rate of 10 °C min^-1^ to recover the crystallinity.

### Characterization

Powder X-ray diffraction (XRD) was performed by a Bruker D2 Phaser using Cu Kα radiation. The morphological characterization was obtained from field-emission SEM (FESEM, JEOL JSM-6700F). Sub-angstrom-resolution high-angle annular dark-field scanning transmission electron microscopy (HAADF-STEM) was conducted with a JEOL JEMARM200F STEM. Scanning transmission electron microscopy (STEM) as well as high-resolution electron energy loss spectroscopy (EELS) analysis were performed on a HITACHI HF5000 operated at 200 kV. The metal content in the catalysts was quantified by inductively coupled plasma-atomic emission spectroscopy (ICP-AES, PerkinElmer). N_2_ adsorption-desorption was conducted by an Autosorb-6 (Quantachrome) at 77 K. Before measurement, the samples were degassed at 200 °C for 5 h. BET surface area was calculated in the *P*/*P*_0_ range of 0.005–0.2. Pore size distribution was obtained by Barrett–Joyner–Halenda (BJH) method using the adsorption branch. Raman spectra were collected on a Renishaw INVIA Reflex Raman spectrometer using 514 nm laser as the excitation source. X-ray photoelectron spectroscopy (XPS) was recorded on a Thermofisher ESCALAB 250Xi photoelectron spectrometer (Thermofisher Scientific). X-ray absorption spectroscopy (XAS) including both X-ray absorption near-edge structure (XANES) and extended X-ray absorption fine structure (EXAFS) at Fe and Ni K-edge were collected in total-fluorescence-yield mode at ambient air at the BL14W1 beamline of the Shanghai Synchrotron Radiation Facility (SSRF). EXAFS analysis was conducted using Fourier transform on k^3^-weighted EXAFS oscillations to evaluate the contribution of each bond to Fourier transform peak.

### Electrochemical measurements

The electrochemical measurements were conducted by an electrochemical workstation (CHI 760E) in a three-electrode configuration using an Ag/AgCl electrode with saturated KCl bridge as the reference and carbon rod as the counter electrode on a rotating disk electrode (RDE) setup (5.0 mm of disk diameter, Pine Inc.). RHE potentials were calculated based on the Nernst equation (*E*_RHE_ = *E*_Ag/AgCl_ + 0.059 × pH + 0.197 V). The catalyst ink was prepared by ultrasonically mixing 5 mg of sample, 0.48 mL of DI water, 0.48 mL of isopropyl alcohol, and 40 μL of 5 wt.% Nafion dispersion. Two microliters of catalyst ink was drop-casted on a glassy carbon RDE. The electrolyte was bubbled with CO_2_ for 30 min, and a flow of CO_2_ was maintained over the electrolyte (0.5 M KHCO_3_) throughout the electrochemical tests. For comparison, CV measurements were also performed in an Ar-saturated electrolyte. Linear sweep voltammograms (LSVs) were collected at a scan rate of 5 mV s^-1^. The Tafel slope was calculated based on the equation (*η* = b log(*j*_*CO*_/*j*_*o*_)), where *η* is the overpotential, b is the Tafel slope, *j*_CO_ is the current density for CO formation, and *j*_*o*_ is the exchange current density.

### H-type electrochemical cell for CO_2_ reduction

The products and Faradaic efficiency of CO_2_ reduction were measured using chronoamperometry at a fixed potential in a H-type electrochemical cell separated by a Nafion membrane. The area of the working electrode is 1 cm^2^. To prepare the working electrode, the catalyst ink (10 mg mL^-1^) was brush-painted directly onto a dry carbon fiber paper (CFP) at 60 °C, giving a catalyst loading of 0.4 mg cm^-2^. The gaseous products were quantified by a gas chromatograph (GC, Agilen 7890) equipped with a flame ionization detector (FID) for CO and CH_4_, and a thermal conductivity detector (TCD) for H_2_ quantification. Ultrapure helium was used as the carrier gas. The average flow rate of CO_2_ was controlled at 10 cc min^-1^ at the inlet of electrochemical cell.

Faradaic efficiency (FE) was calculated by FE_*i*_ = *Q*_*i*_*/Q*_total_, where *i* represents CO and H_2_. *Q*_*i*_ and *Q*_total_ can be obtained from the following equations: *Q*_*i*_ = *Z*_*i*_ × *F* × *N*_*i*_ and *Q*_*total*_ = *I* × *t*, where *Z*_*i*_ is the number of electrons required to produce *i* molecule, which is 2 for CO and 2 for H_2_,; *F* is Faradaic constant; *I* is the average current in a period *t* of electrocatalysis. Based on the GC data and ideal gas law: *N*_*i*_ = *N*_total_ × *V*_*i*_, *N*_total_ = (*P*_*o*_ × *V*_*o*_)*/*(*R* × *T*), and *V*_*o*_ = *G* × *t*, where *N*_total_ and *N*_*i*_ are the mole numbers of all gases and product *i* in the GC sampling loop, respectively; *V*_*i*_ and *V*_*o*_ are the volume ratio of product *i* and the volume of the GC sampling loop, respectively; *P*_*o*_ is the atmospheric pressure; *T* is the reaction temperature (298 K); *R* is ideal gas constant; *G* is the volumetric flow rate; *t* is the time for gas to fill the GC sampling loop.

The turnover frequency (TOF) for the CO_2_ reduction reaction was evaluated based on the 2-electron pathway (as shown in the following formula).

1 $${\mathrm{TOF}}=\frac{{\mathrm{Turnover}}\,{\mathrm{number}}\,({\mathrm{TON}})\,{\mathrm{for}}\,{\mathrm{CO}}\,{\mathrm{formation/geometric}}\,{\mathrm{area}}}{{\mathrm{Number}}\,{\mathrm{of}}\,{\mathrm{activesites/geometric}}\,{\mathrm{area}}}$$

2 $${\mathrm{TON}} =\frac{Q\times {\mathrm{F}}{{\mathrm{E}}}_{{\mathrm{CO}}}}{2F}=\frac{J\times t\times {\mathrm{F}}{{\mathrm{E}}}_{{\mathrm{CO}}}}{2F}=\left(\frac{51.1\times {10}^{-3}\times 3600\times 0.945}{2\times 96,485.3}\right)\times 6.02\times {10}^{23}\\ =5.42\times {10}^{20}{\mathrm{c}}{{\mathrm{m}}}^{-2}{{\mathrm{h}}}^{-1}$$

where *Q* is the total charge, *J* and *FE*_CO_ are current density and Faradaic efficiency of CO formation at the overpotential of 0.68 V, respectively. *F* is Faraday constant. Catalyst loading on glassy carbon rotating disk electrode is 0.01 mg cm^-2^. Number of active sites per cm^2^ of NiFe-DASC can be calculated as following:

3 $$N={\mathrm{Avogadro}}\,{\mathrm{constant}}\times \left(\frac{{\mathrm{Weight}}\,{\mathrm{of}}\,{\mathrm{activesites}}}{{\mathrm{Molecular}}\,{\mathrm{weight}}\,{\mathrm{of}}\,{\mathrm{activesites}}}\right)$$

$$\,\,\,\,\,\,\,\,\,\,\,\,\,\,\,=6.02\times {10}^{23}\times \left[\frac{(1\times {10}^{-4})\times (3.24\times {10}^{-2})}{54}\right]=3.6\times {10}^{16}c{m}^{-2}.$$

F or NiFe-DASC, TOF = ${\mathrm{TON}}/N=5.42\times \frac{{10}^{20}}{3.6}\times {10}^{16}=15055{{\mathrm{h}}}^{-1}$. For Fe-SAC and Ni-SAC, *TOF*s are 1731 h^-1^ and 10179 h^-1^, respectively.

### Rechargeable Zn-CO_2_ battery

The rechargeable Zn-CO_2_ battery was tested using a home-made cell in a double-electrolyte system separated by two bipolar membranes. The air cathode was prepared using a carbon paper (1 × 1 cm^2^) with catalysts coated on the liquid-facing side. The catalyst ink (10 mg of catalysts dispersed in a mixture of 425 μL water, 425 μL ethanol and 50 μL 5 wt.% Nafion solution) was deposited onto carbon paper with a loading amount of 10 mg cm^-2^. The anode is a zinc plate (2 × 3 cm, thickness 2 mm). Catholyte is 2 M KCl and anolyte is 2 M KOH + 0.02 M Zn(CH_3_COO)_2_. CO_2_ was controlled by a mass flowmeter with a rate of 10 mL min^-1^. The discharge current was controlled to be 1–10 mA cm^-2^ in the galvanostatic discharge tests. The liquid and gaseous products were analyzed by on-line gas chromatography and off-line high-performance liquid chromatography.

### Theoretical calculations

First-principles calculations were performed using the Vienna Ab-Initio Simulation Package based on spin-polarized Perdew–Burke–Ernzerhof functional^42,43^. Van der Waals correction of Grimme scheme (D2) was used to improve the description of the dispersion interaction between adsorbates and substrates^44–46^. A vacuum thickness of over 15 Å was added in the z direction to avoid unphysical interactions between periodic images. All calculations were conducted with a plane wave cutoff of 500 eV and a 5 × 5 × 1 *k*-mesh, which is enough to ensure accurate results.

OER in alkaline solution proceeds via four successive reaction steps:

4 $${{\rm{OH}}}^{-}+\ast \to \ast {{\rm{OH}}+{\rm{e}}}^{-}$$

5 $${{\rm{OH}}}^{-}+\ast {\rm{OH}}\to \ast {{\rm{OH}}}_{2}{\rm{O}}({l}){+{\rm{e}}}^{-}$$

6 $${{\rm{OH}}}^{-}+\ast {\rm{O}}\to \ast {{\rm{OOH}}+{\rm{e}}}^{-}$$

7 $${{\rm{OH}}}^{-}+\ast {\rm{OOH}}\to \ast +{{\rm{O}}}_{2}({g}){{\rm{H}}}_{2}{\rm{O}}({l}){+{\rm{e}}}^{-}$$

The electroreduction of CO_2_ to CO proceeds via three elementary steps according to previous studies^47^.

8 $${{\rm{CO}}}_{2}({\rm{g}}){+\ast +{\rm{H}}}^{+}{+{\rm{e}}}^{-}\to \ast {\rm{COOH}}$$

9 $${\ast {\rm{COOH}}+{\rm{H}}}^{+}{+{\rm{e}}}^{-}\to \ast {{\rm{CO}}+{\rm{H}}}_{2}{\rm{O}}({\rm{l}})$$

10 $$\ast {\rm{CO}}\to \ast +{\rm{CO}}$$

*OH, *O, and *OOH are the involved reaction intermediates in OER; O_2_(*g*) and H_2_O(*l*) are gas-phase O_2_ and liquid-phase H_2_O, respectively. *COOH and *CO are the reaction intermediates in CO_2_RR. For each elementary step, the associated Gibbs free energy change (Δ*G*) was calculated by

11 $$\Delta {G}=\Delta {E}+\Delta {\rm{ZPE}}-{T}\Delta {S}$$

Δ*E, Δ*ZPE, and Δ*S* are total energy, zero-point energy and entropy changes relative to the initial state. ZPE was calculated from the vibrational frequencies^47^, while entropy was taken from the NIST-JANAF thermodynamics table for gaseous molecules^48^. Solvation effects were considered according to previous studies^49,50^: intermediates of *COOH and *CO were stabilized, respectively, by 0.25 and 0.10 eV. A correction of −0.51 eV was made to the energetic gas-phase due to the Perdew–Burke–Ernzerhof functional^51^. For each step involving an electron transfer from OH^−^ to e^−^ in OER, the corresponding free energy change was calculated based on the standard hydrogen electrode method proposed by Nørskov et al.^52^.

12 $${{\rm{OH}}}^{-}{-{\rm{e}}}^{-}\to {{\rm{H}}}_{2}{{\rm{O}}-1/{\rm{2H}}}_{2}$$

13 $${G}({{\rm{OH}}}^{-})-{G}({{\rm{e}}}^{-})={G}({{\rm{H}}}_{2}{\rm{O}})-{G}({1/{\rm{2H}}}_{2})+{{k}}_{{B}}{T}{\rm{ln10}}\cdot {\rm{pH}}$$

The last term is a correction to the Gibbs free energy of OH^−^ anion at a certain pH value. Here, the temperature and pH is set to be 298.15 K and 14, respectively. *k*_*B*_ is the Boltzmann constant. The calculated overpotential is independent of the choice of pH value^53^.

The Gibbs free energy of O_2_ was calculated from H_2_O → 1/2O_2_+H_2_ using experimentally determined reaction energy (2.46 eV) to avoid the well-known errors of describing its high-spin ground state in first-principles simulations. For liquid H_2_O, temperature (*T*) and entropy (*S*) were calculated based on gas phase with a fugacity of 0.035 atm (the vapor pressure of liquid water at room temperature) through the equation^54^.

14 $${{\rm{TS}}={\rm{TS}}}_{0}+{k_{\mathrm{B}}T{\mathrm{ln}}}({p_0}/{p})$$

where *p* is partial pressure (0.035 atm) and *p*_0_ is 1 atm; *S*_0_ is the gas-phase entropy. Finally, the OER overpotential was determined by

15 $${{\rm{\eta }}}_{{\rm{OER}}}={{\mathrm{\varDelta}}} {G}_{{{\max }}}/e-{U}_{0}$$

where Δ*G*_max_ is the maximum Gibbs free energy change of the four reaction steps; *U*_0_ (0.402 V) is the equilibrium potential at pH = 14 and *T* = 298.15 K.

## Supplementary information

Supplementary Information

## Data Availability

The data that support the findings of this study are available from the corresponding authors upon reasonable request.
