# Supplementary material for: Orbital coupling of hetero-diatomic nickel-iron site for bifunctional electrocatalysis of CO2 reduction and oxygen evolution
Source: Nat Commun. 2021 Jul 2;12:4088. doi: 10.1038/s41467-021-24052-5 (PMC8253796; doi:10.1038/s41467-021-24052-5)
Supplement: Supplementary file 1 — Supplementary Information [file 41467_2021_24052_MOESM1_ESM.pdf]

Supplementary information

Orbital coupling of hetero-diatomic nickel-iron site for bifunctional electrocatalysis of CO_2 reduction and oxygen evolution

Zhiping Zeng[1,2], Li Yong Gan[3], Hong Bin Yang[4]✉, Xiaozhi Su[5], Jiajian Gao[2], Wei Liu[6], Hiroaki Matsumoto[7], Jun Gong[2], Junming Zhang[2], Weizhen Cai[2], Zheye Zhang[2], Yibo Yan[8], Bin Liu[2]✉ & Peng Chen[2]✉

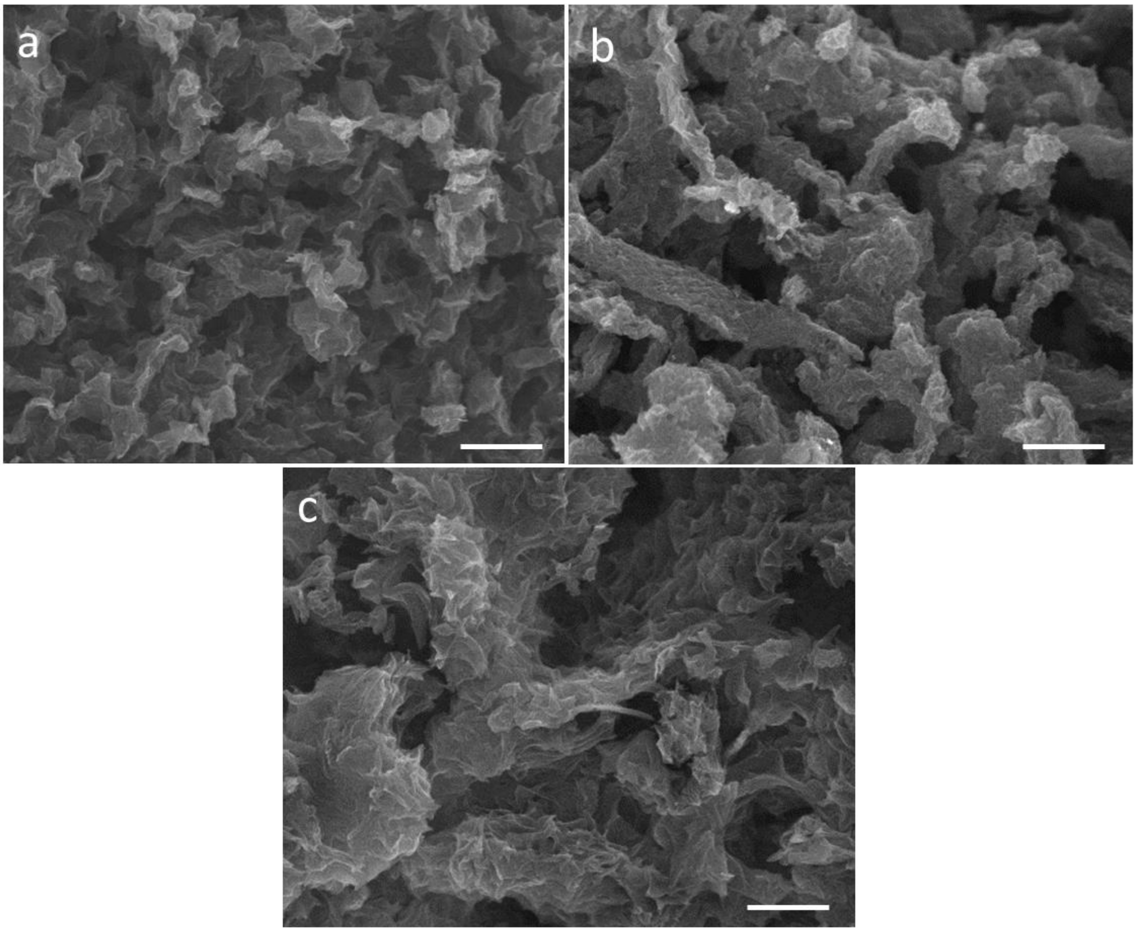

Supplementary Figure 1. SEM image of (a) Ni-SAC, (b) Fe-SAC, and (c) NiFe-DASC. Scale bar: 2 μm.

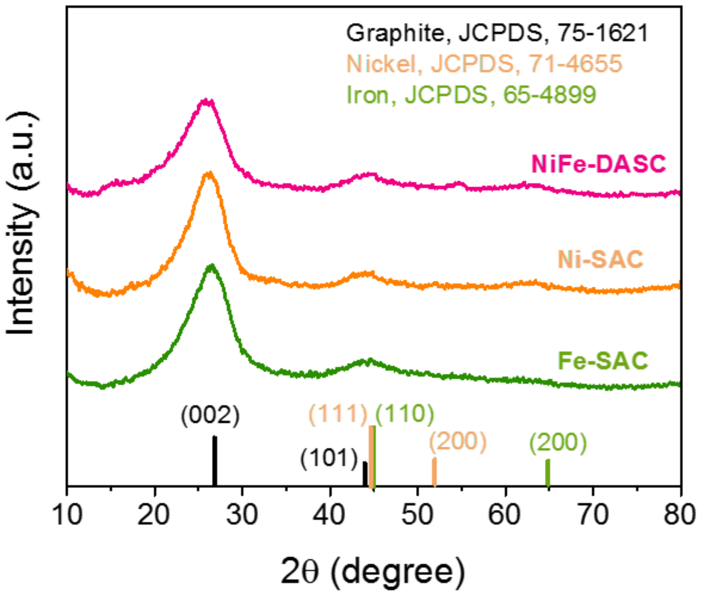

Supplementary Figure 2. XRD patterns of NiFe-DASC, Ni-SAC, and Fe-SAC.

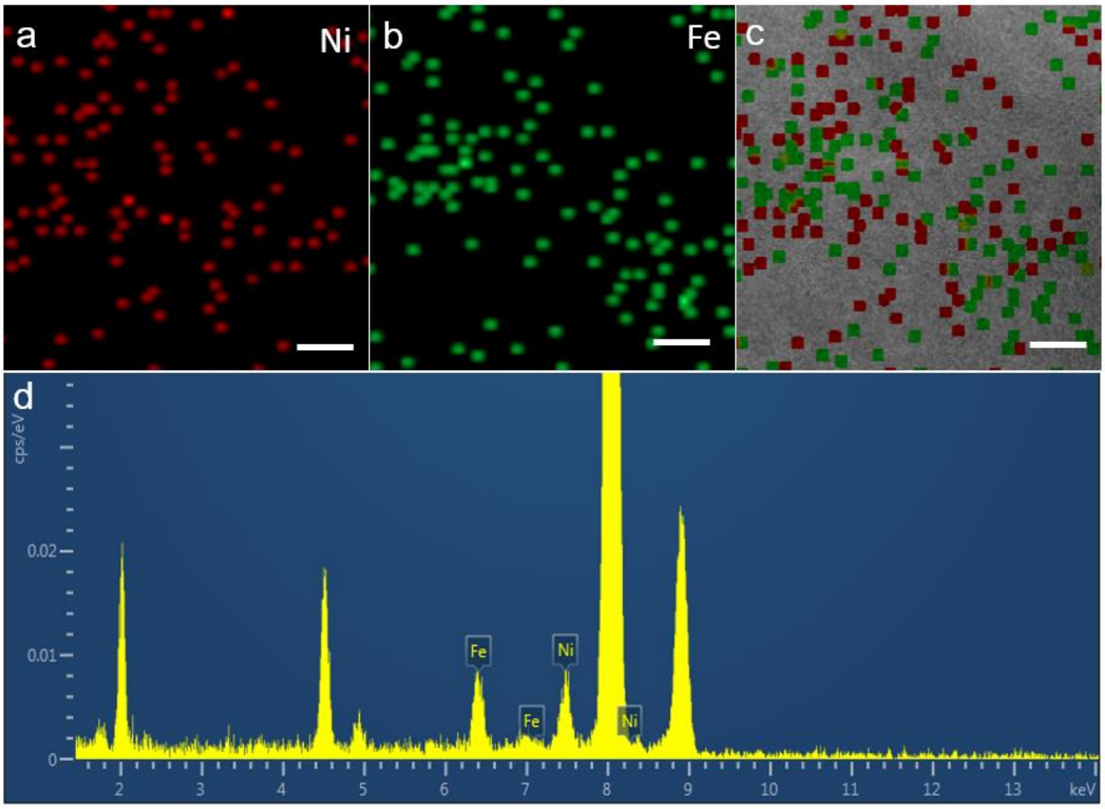

Supplementary Figure 3. (a-c) EDS elemental mapping and (d) EDS elemental spectrum of NiFe-DASC.

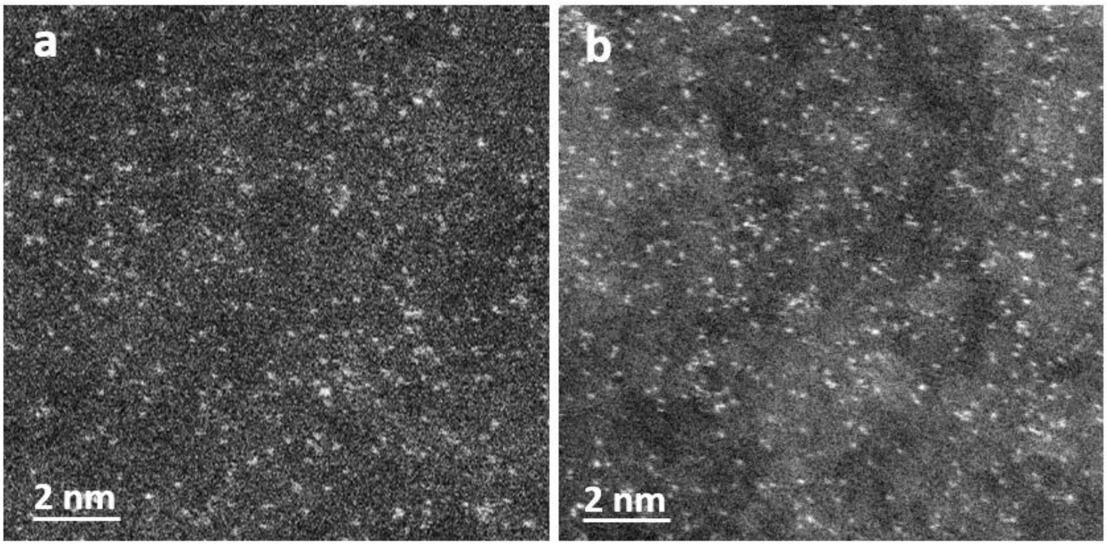

Supplementary Figure 4. HAADF-STEM images of (a) Ni-SAC and (b) Fe-SAC.

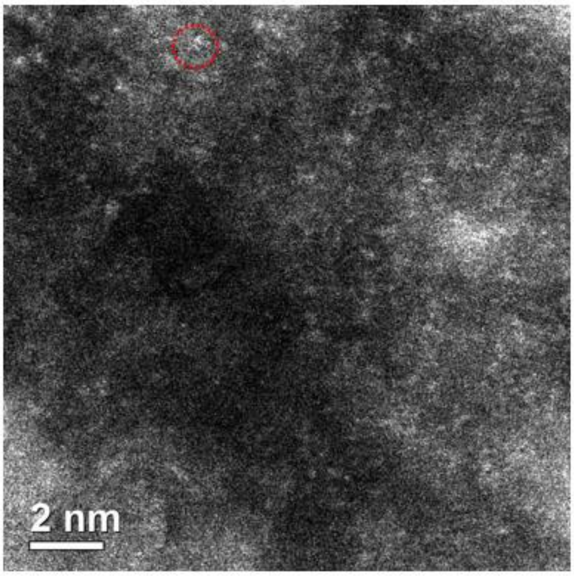

Supplementary Figure 5. The HAADF-STEM image of NiFe-DASC with the area (red circle) acquired for EELS.

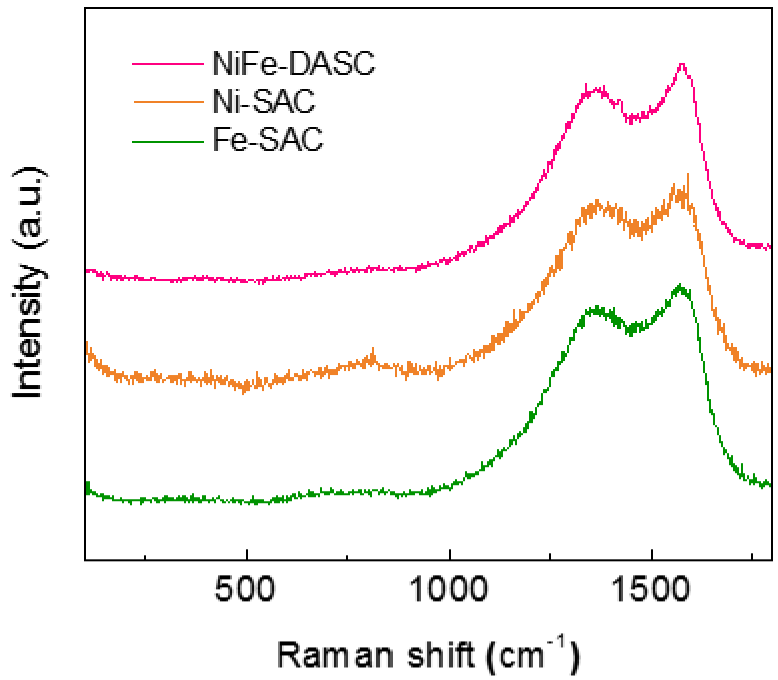

Supplementary Figure 6. Raman spectra of NiFe-DASC, Fe-SAC, and Ni-SAC.

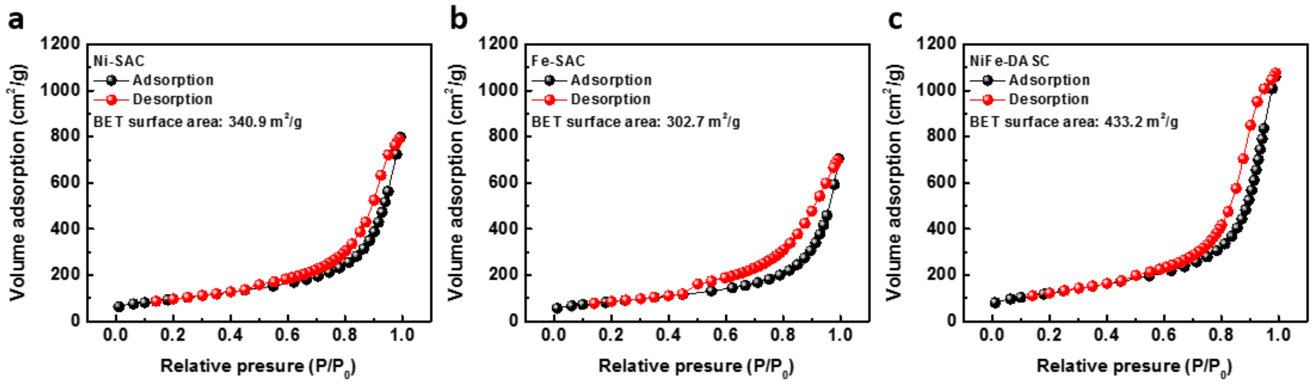

Supplementary Figure 7. Nitrogen adsorption isotherms of (a) Ni-SAC, (b) Fe-SAC, and (c) NiFe-DASC measured at 77 K. BET surface areas of Ni-SAC, Fe-SAC, and NiFe-DASC are determined to be 340.9, 302.7, and 433.2 m^2 g^{-1}, respectively.

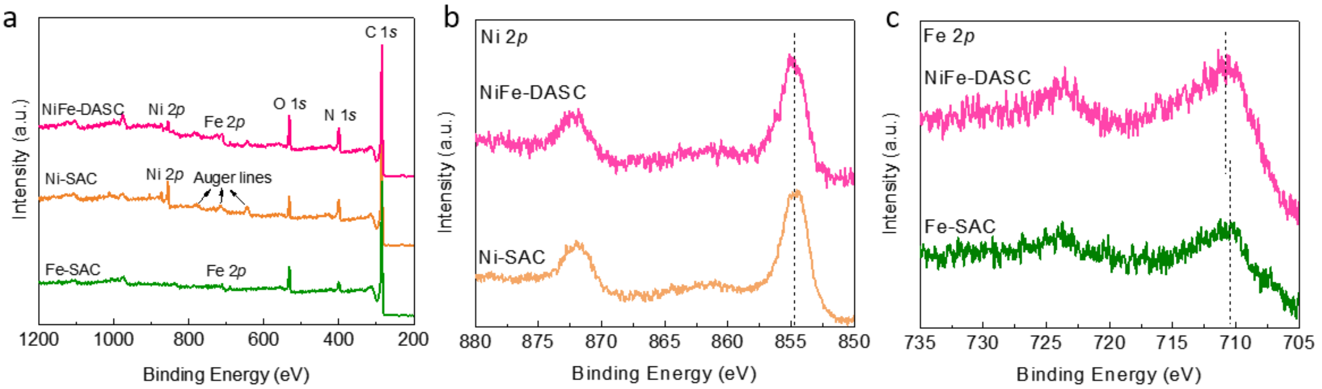

Supplementary Figure 8. (a) XPS survey of NiFe-DASC, Ni-SAC, and Fe-SAC. High resolution XPS of (b) Ni 2*p* spectra in NiFe-DASC and Ni-SAC, (c) Fe 2*p* spectra in NiFe-DASC and Fe-SAC.

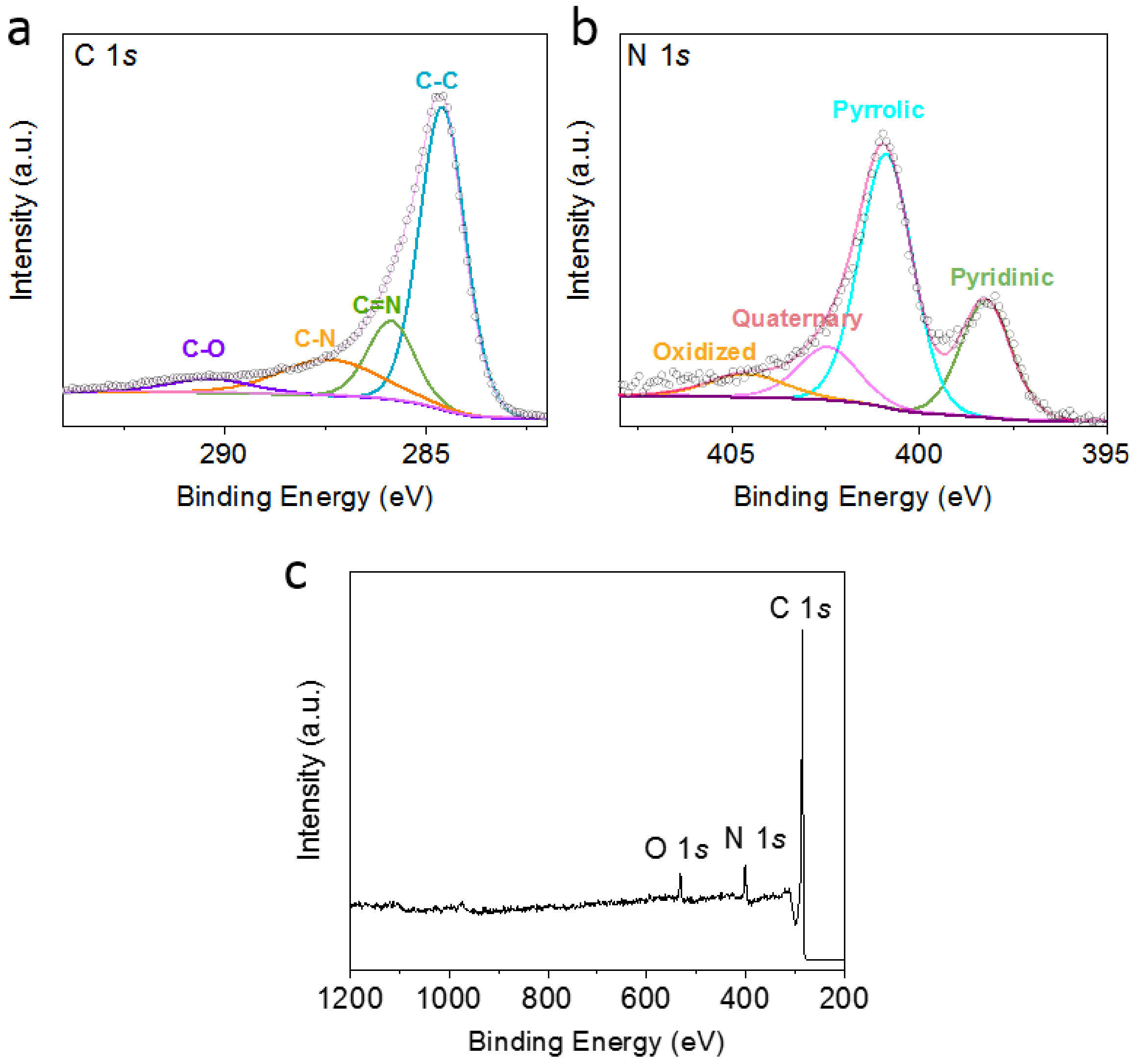

Supplementary Figure 9. High-resolution XPS spectra of (a) C 1*s* and (b) N 1*s*, and XPS survey spectrum of N-doped graphene.

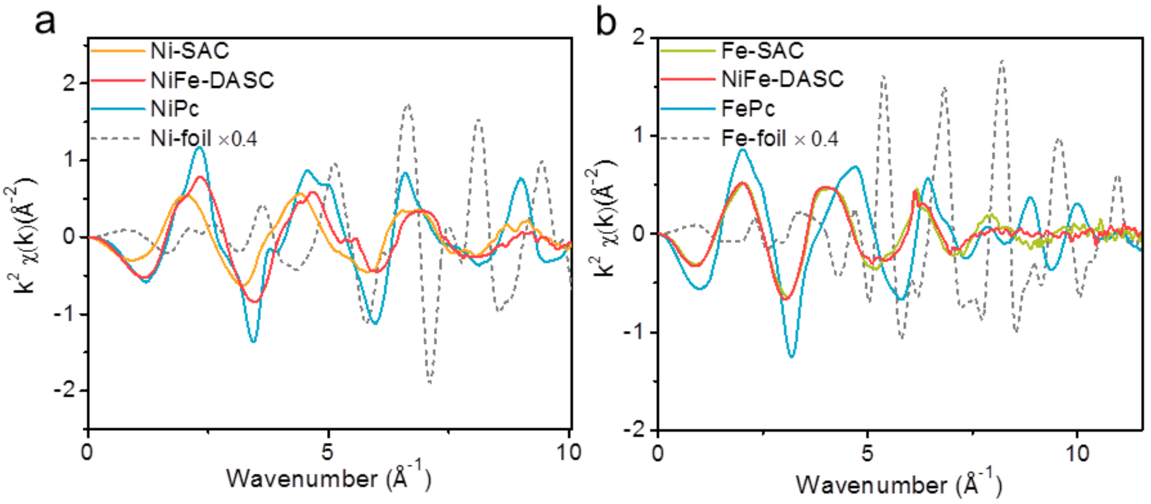

Supplementary Figure 10. k^2-weighted k-space spectra of (a) Ni-SAC, NiFe-DASC, nickel (Ⅱ) phthalocyanine (NiPc), and Ni-foil, (b) Fe-SAC, NiFe-DASC, iron (Ⅱ) phthalocyanine (FePc), and Fe-foil.

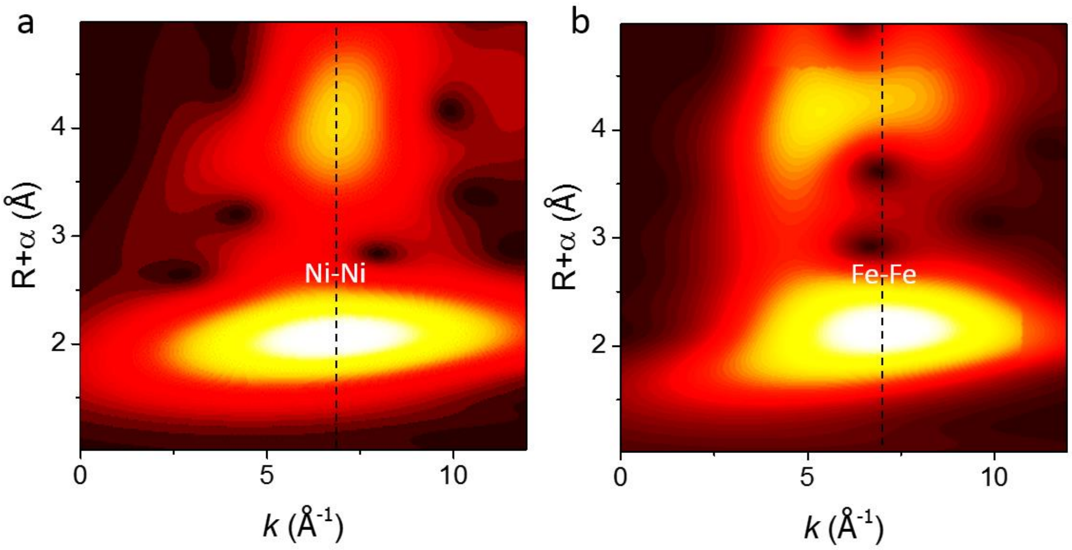

Supplementary Figure 11. Wavelet transform (WT)-EXAFS of k^2-weighted k-space spectra of (a) Ni for Ni foil and (b) Fe for Fe foil.

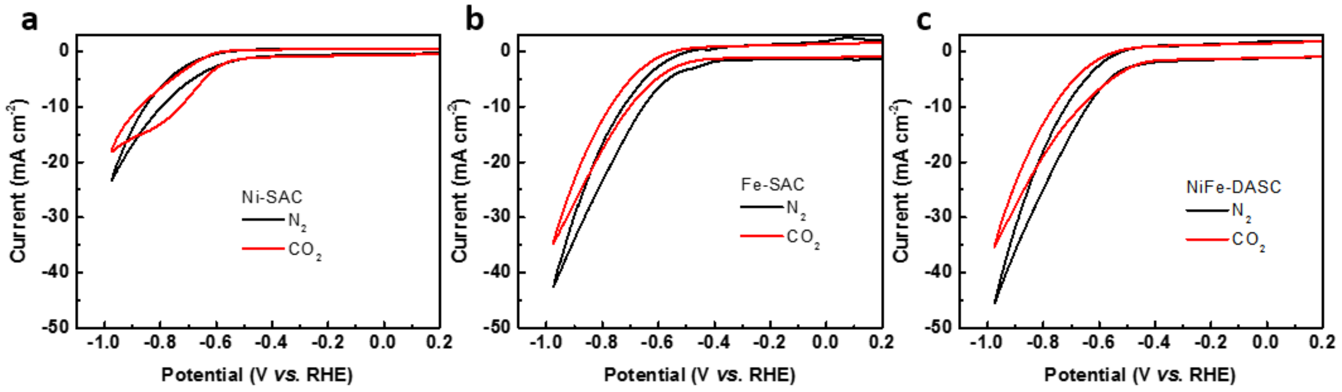

Supplementary Figure 12. Cyclic voltammograms of (a) Ni-SAC, (b) Fe-SAC, and (c) NiFe-DASC on GC electrode in N_2 and CO_2 saturated 0.5 M $KHCO_3$ at a scan rate of 50 mV s^{-1}. The cathodic peaks at ~0.68 V and ~0.61 V (vs RHE) were observed in the CV curves of Ni-SAC and NiFe-DASC, separately, suggesting their good electrochemical activities for CO_2RR.

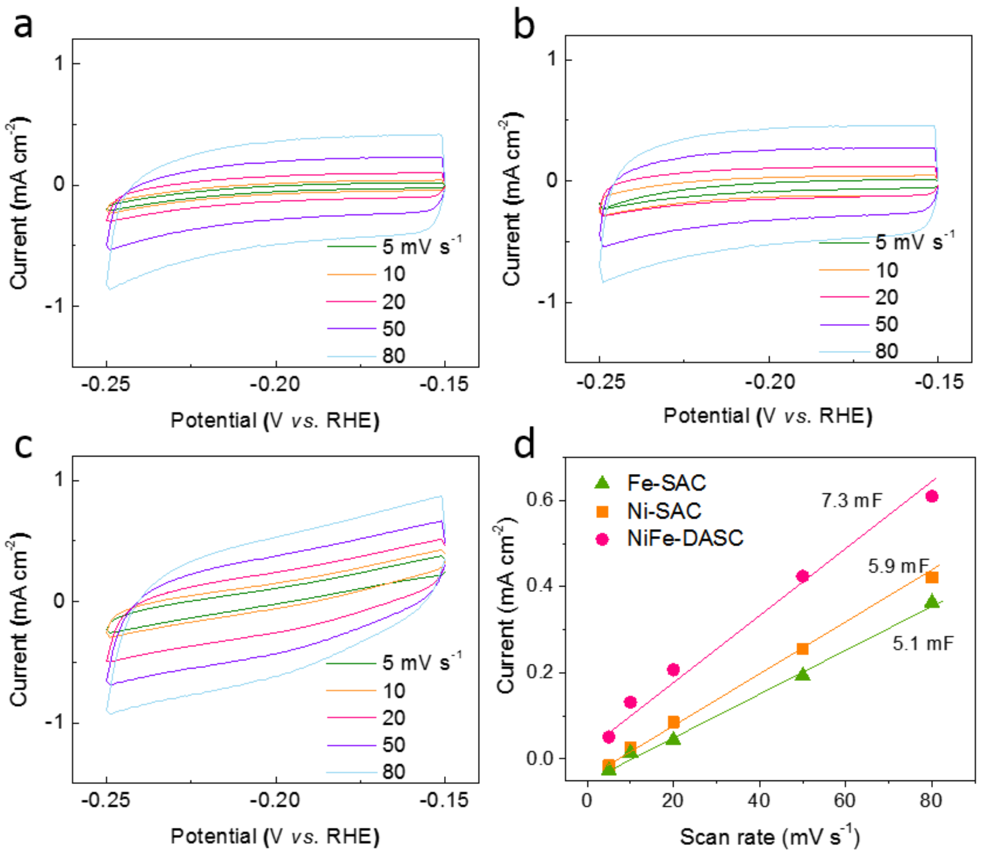

Supplementary Figure 13. Determination of electrochemically active surface area (ECSA). (a-c) CV curves of Fe-SAC, Ni-SAC, and NiFe-DASC in the capacitance region at different scan rates. (d) The capacitance current at -0.2 V vs. RHE as a function of scan rate.

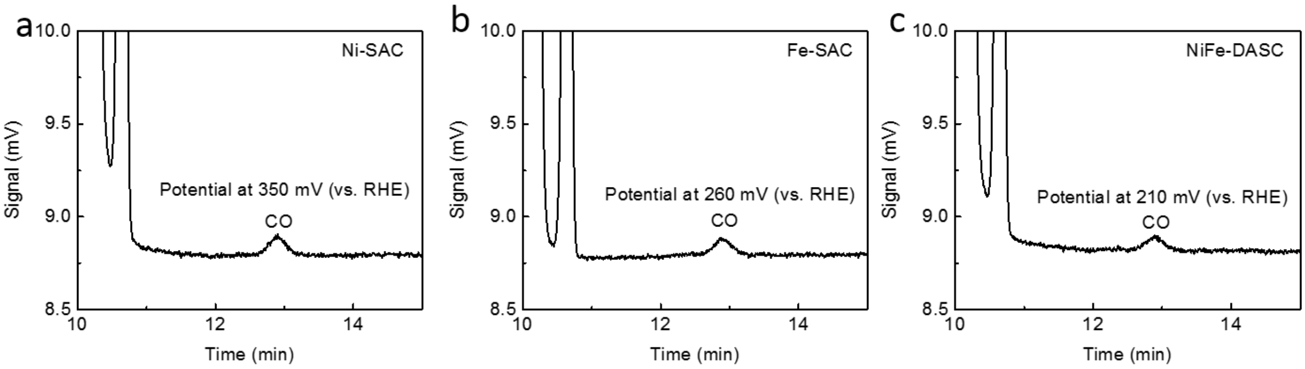

Supplementary Figure 14. Flame ionization detector (FID) signal from the electrons at a potential of (a) 350 mV for Ni-SAC, (b) 260 mV for Fe-SAC, and (c) 210 mV for NiFe-DASC in 0.5 M CO_2-saturated $KHCO_3$ aqueous solution.

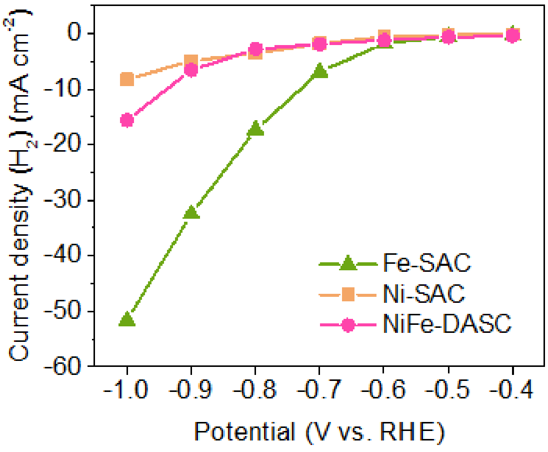

Supplementary Figure 15. Partial current density of H_2.

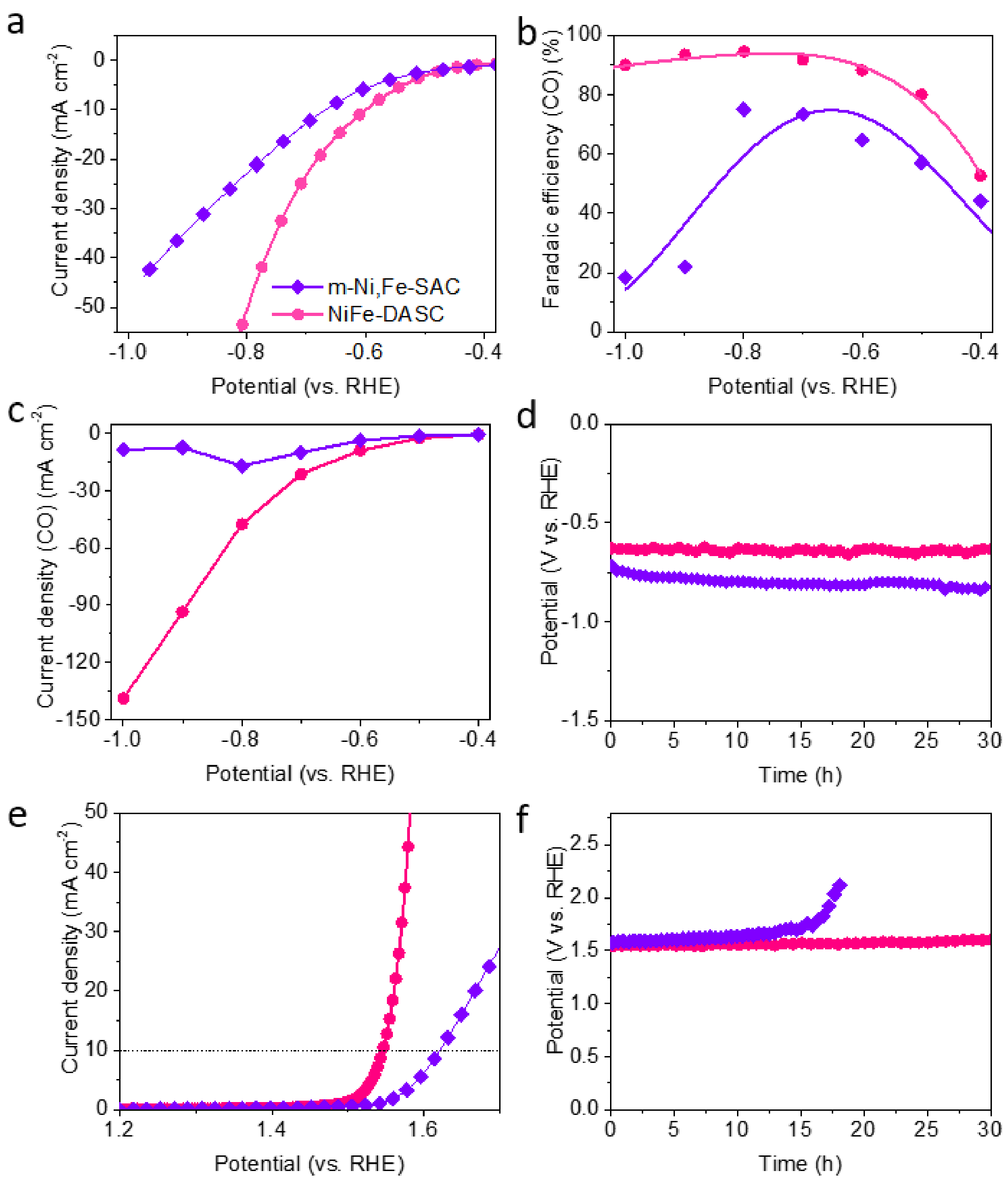

Supplementary Figure 16. (a) Linear sweep voltammograms of physically mixed Ni- and Fe-SAC (m-Ni,Fe-SAC), and NiFe-DASC acquired on a rotating disc electrode at a rotation speed of 1600 r.p.m. and a scan rate of 5 mV s^{-1}. Catalyst loading is 0.1 mg cm^{-2} and electrolyte is CO_2-saturated 0.5 M $KHCO_3$ solution. (b) Faradaic efficiencies of CO generation at various potentials (c) Partial current density of CO (j_{CO}) in potentiostatic electrolysis. (d) Potential-time response for CO_2RR at a current density of 10 mA cm^{-2}. (e) LSV curves for OER in O_2-saturated 1.0 M KOH at a rotation speed of 1600 r.p.m and a scan rate of 1 mV s^{-1}. (f) Potential-time response for OER at a current density of 10 mA cm^{-2}.

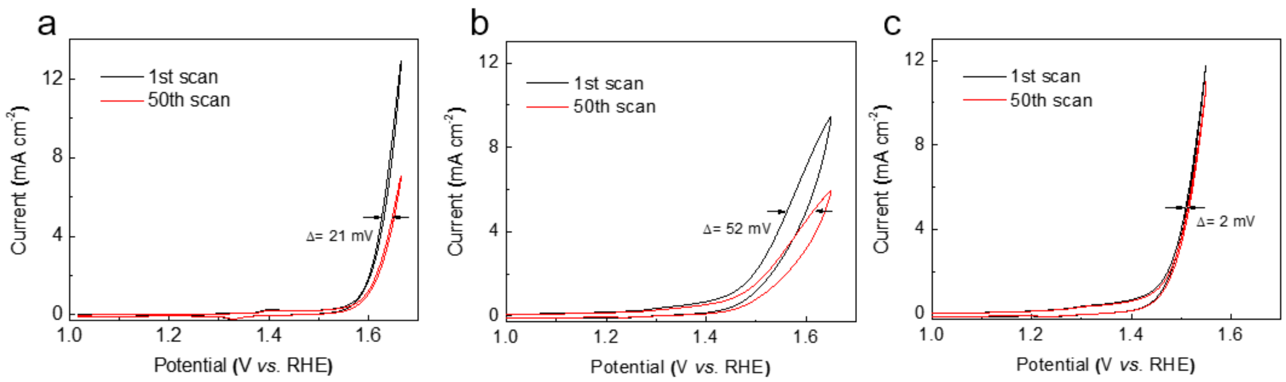

Supplementary Figure 17. Cyclic voltammograms of (a) Ni-SAC, (b) Fe-SAC, and (c) NiFe-DASC on GC electrode in O_2 saturated 1.0 M KOH at a scan rate of 50 mV s^{-1}.

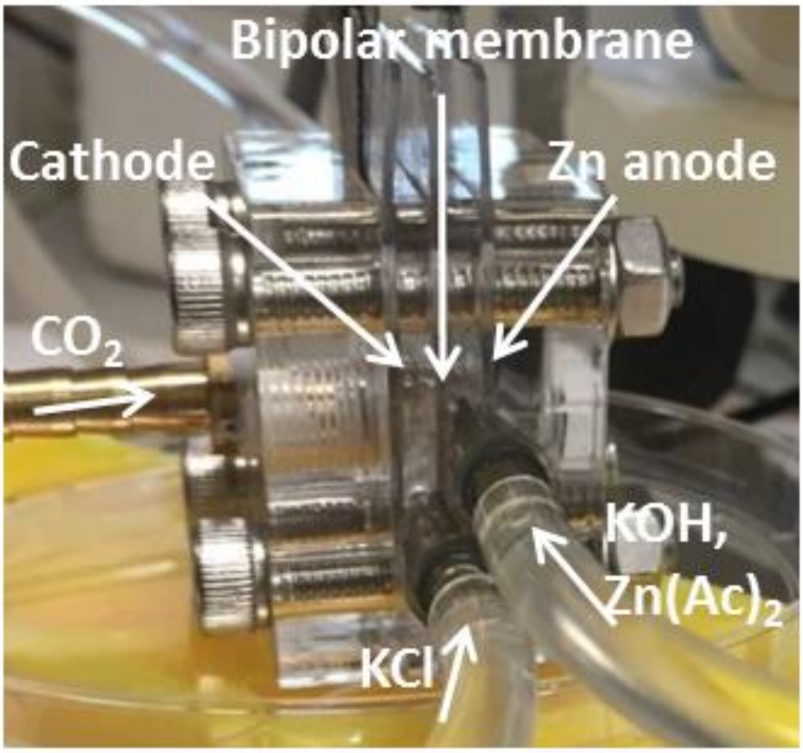

Supplementary Figure 18. Photograph of Zn-CO_2 battery cell.

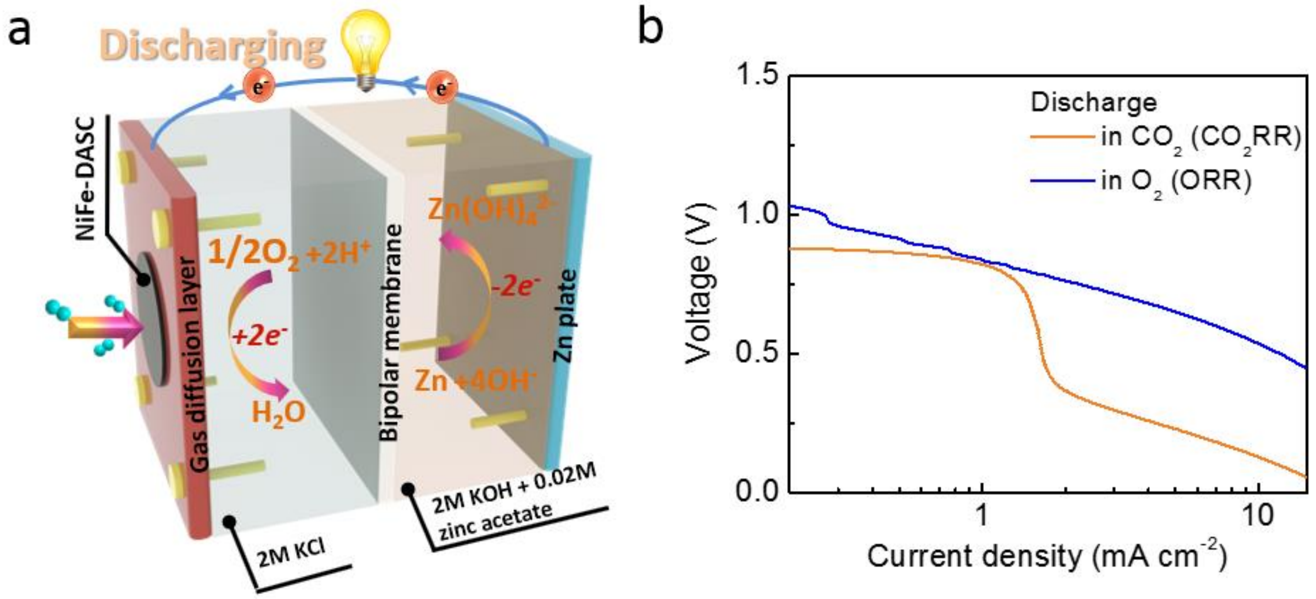

Supplementary Figure 19. (a) Schematic illustration of Zn-O_2 battery. (b) Discharge polarization curves in different gases (CO_2 or O_2) at a scanning rate of 10 mV s^{-1}.

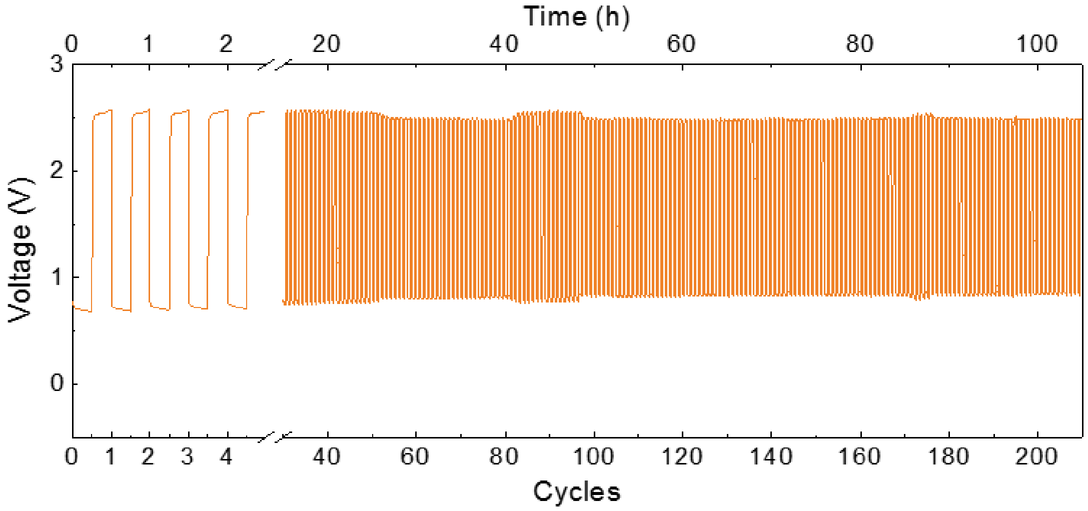

Supplementary Figure 20. Galvanostatic discharge-charge cycling curves at the current density of 1 mA cm^{-2} for 220 cycles.

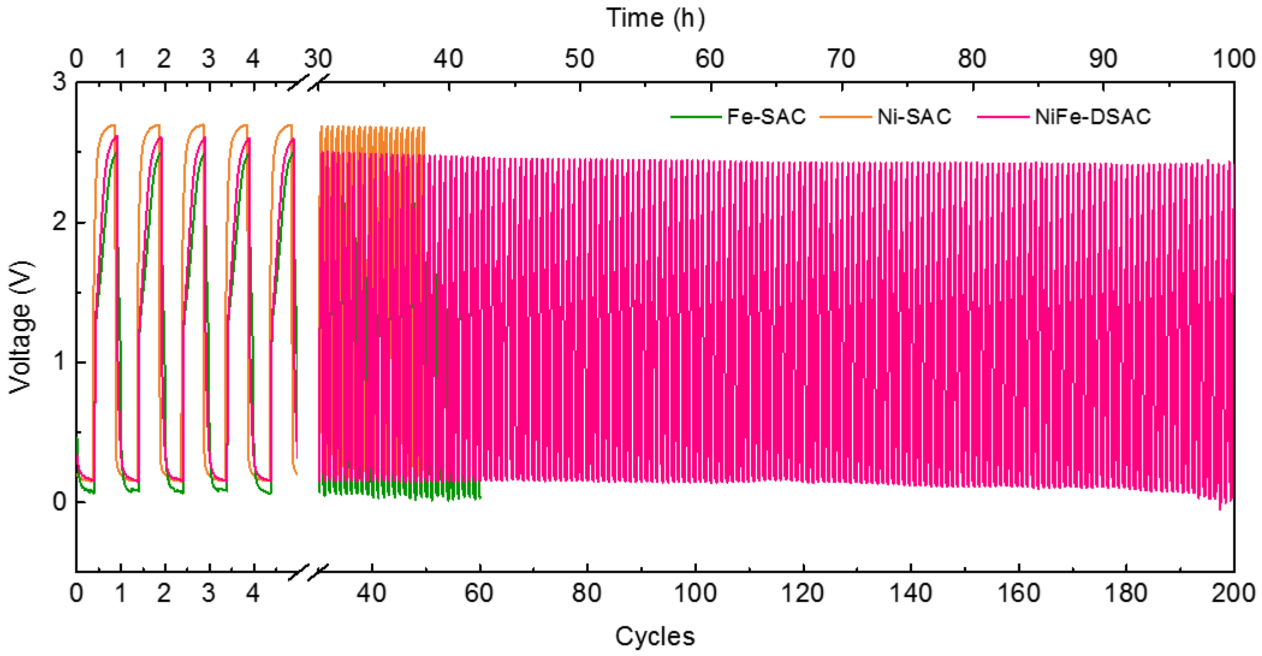

Supplementary Figure 21. Galvanostatic discharge-charge cycling curves at the discharge current of 5 mA cm^{-2} and charge current of 2 mA cm^{-2}.

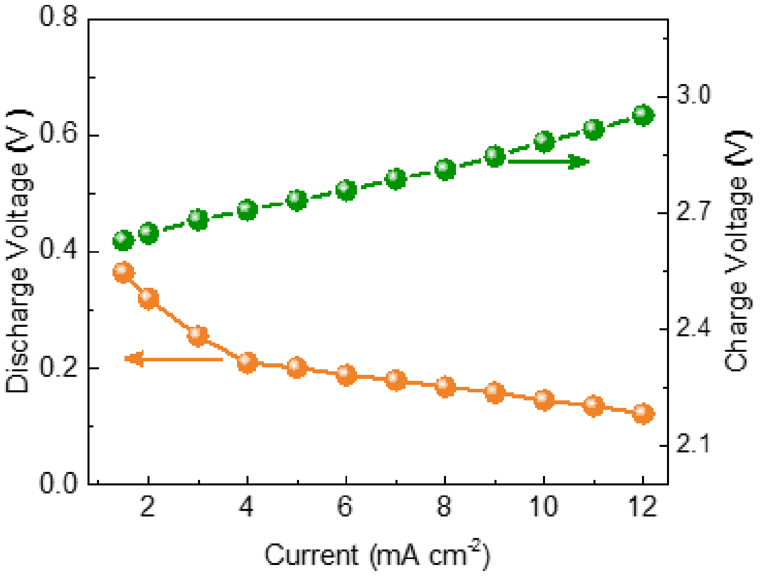

Supplementary Figure 22. Discharge and charge voltage profiles from 1.5 to 12 mA cm^{-2}.

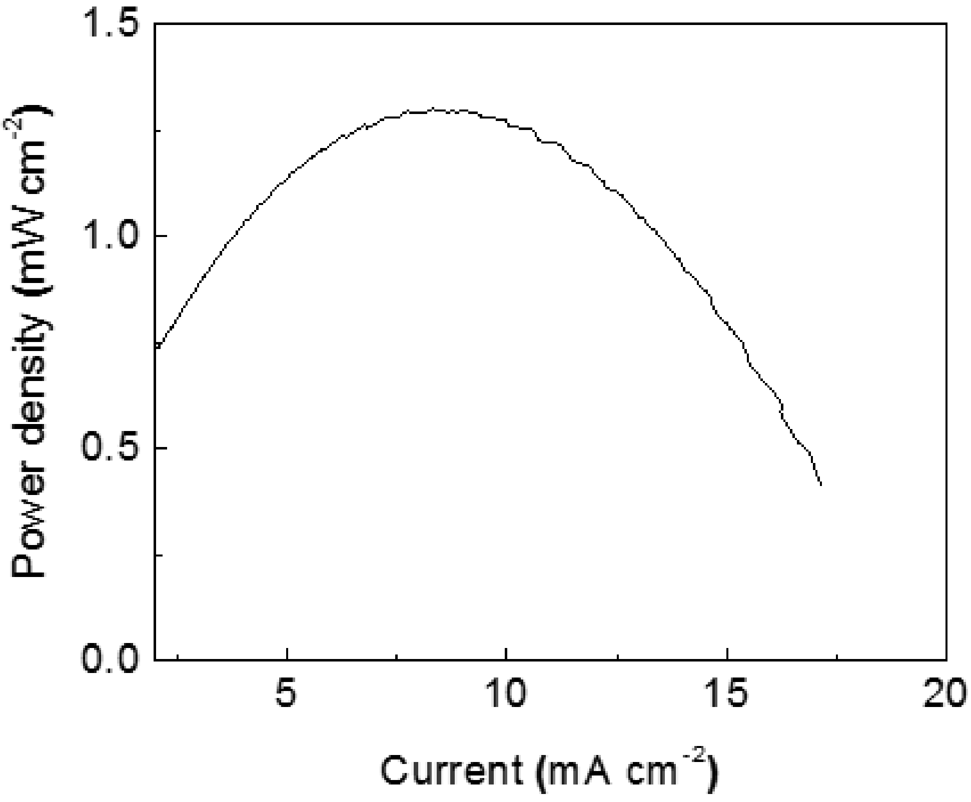

Supplementary Figure 23. Power density plot of the Zn-CO_2 battery cell.

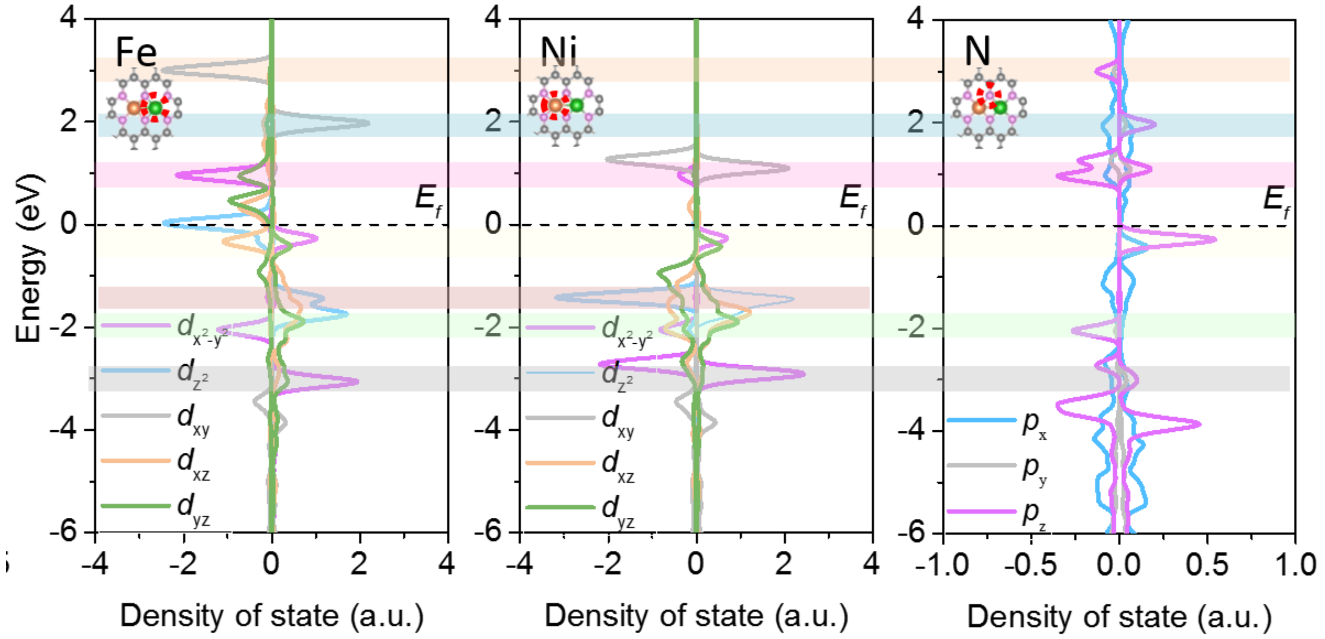

Supplementary Figure 24. Density of state of metals (Fe and Ni) *d* and nitrogen *p* orbitals in NiFe-DASC.

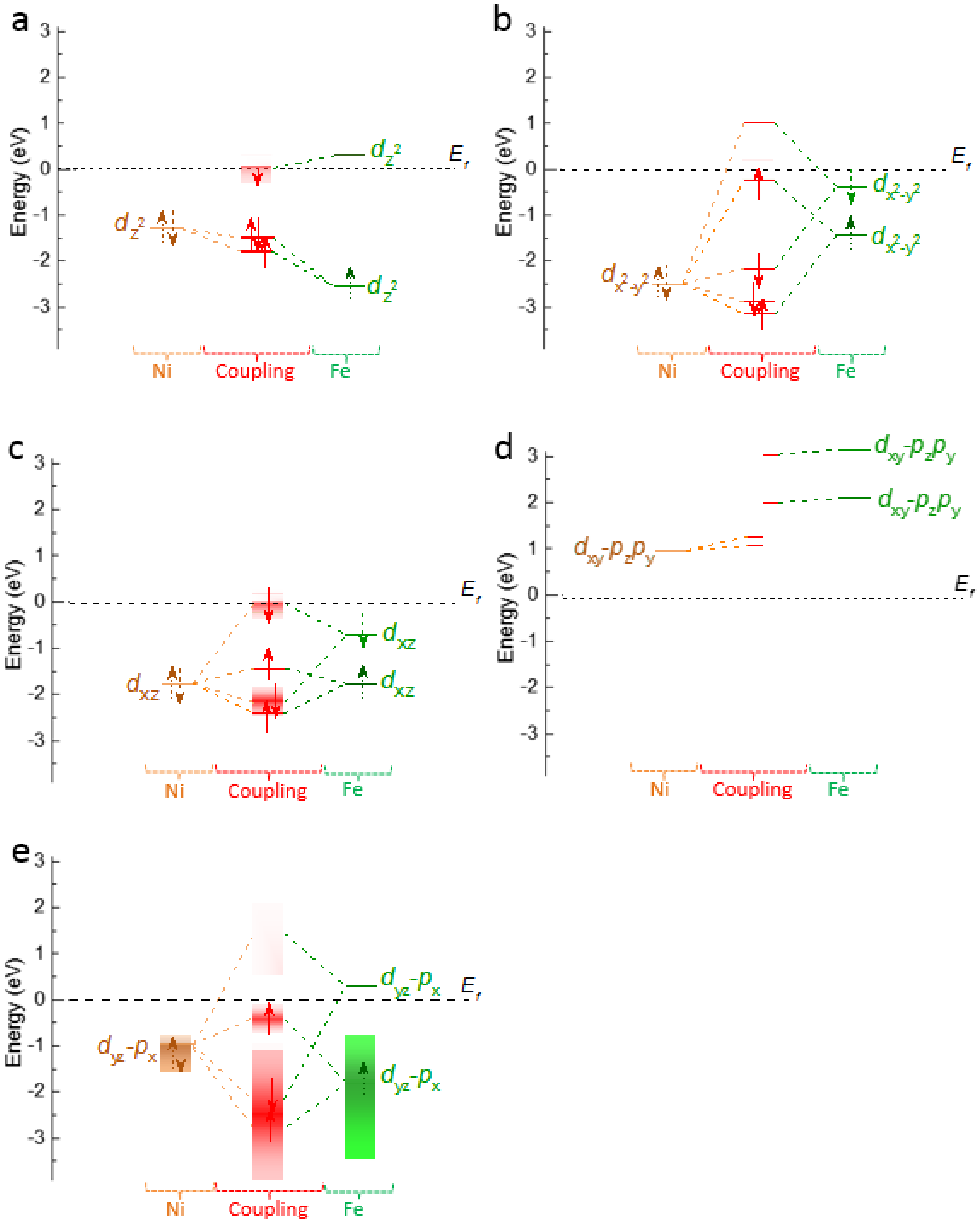

Supplementary Fig. 25. The energy level of (a) d_{z^2}, (b) $d_{x^2-y^2}$, (c) d_{xz}, (d) d_{xy}-p_zp_y, and (f) d_{yz}-p_x of active site in NiFe-DASC. The shade of color in (a, c, e) refers to the probability densities of spin-up/spin-down electrons.

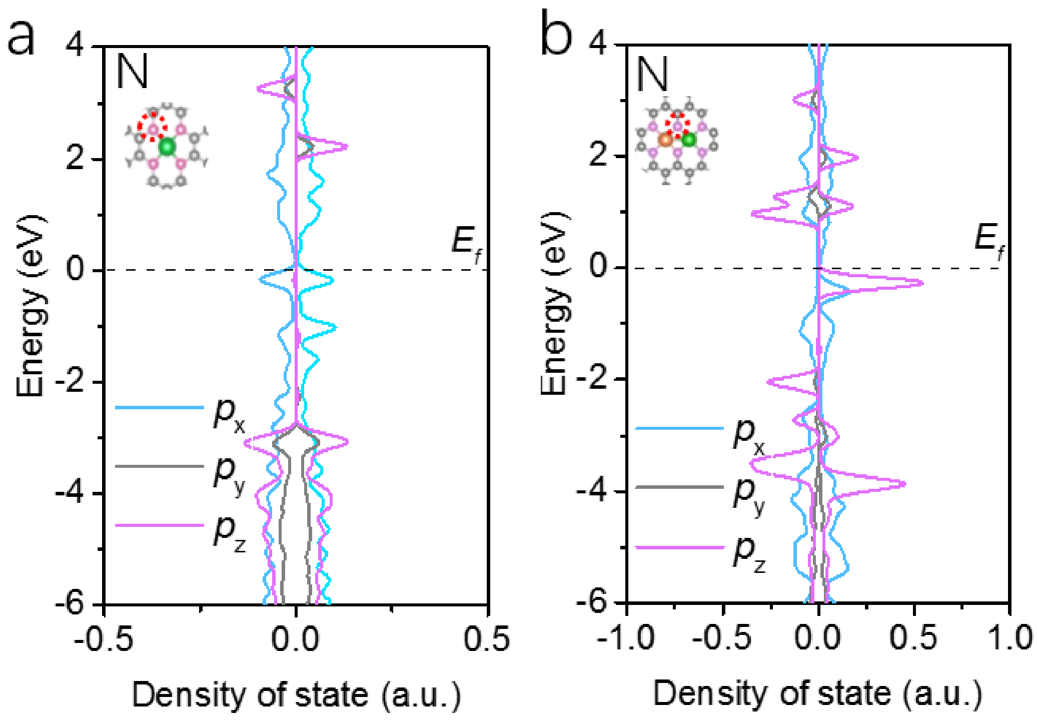

Supplementary Figure 26. Density of state of N 2*p* for (a) Fe-SAC and (b) NiFe-DASC.

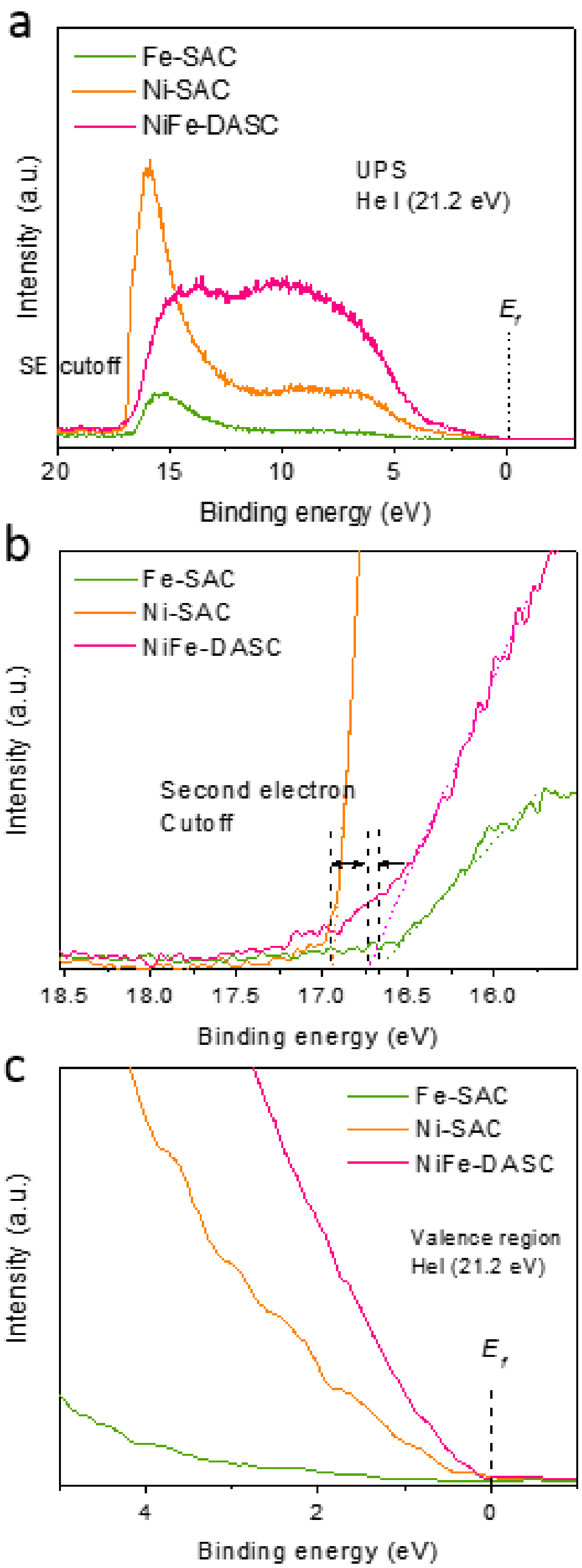

Supplementary Figure 27. (a) Ultraviolet photoelectron spectroscopy (UPS) valence band spectra of Fe-SAC, Ni-SAC, and NiFe-DASC. UPS valence band spectra in (b) the second electron (SE) cutoff region and (c) near Fermi level region.

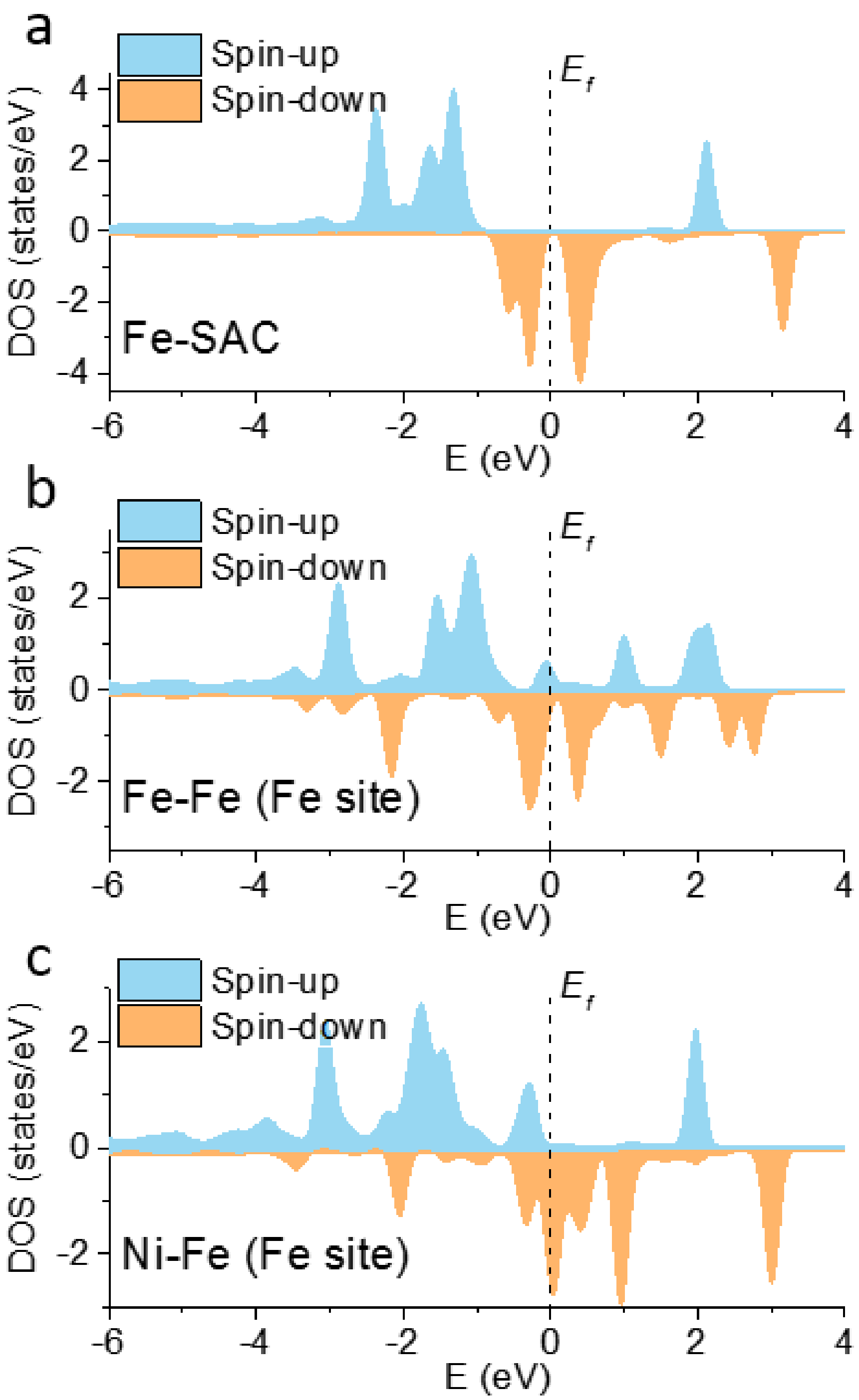

Supplementary Figure 28. Density of states (DOS) of active center in the (a) Fe-SAC, (b) Fe-Fe DASC and (c) Ni-Fe DASC configuration.

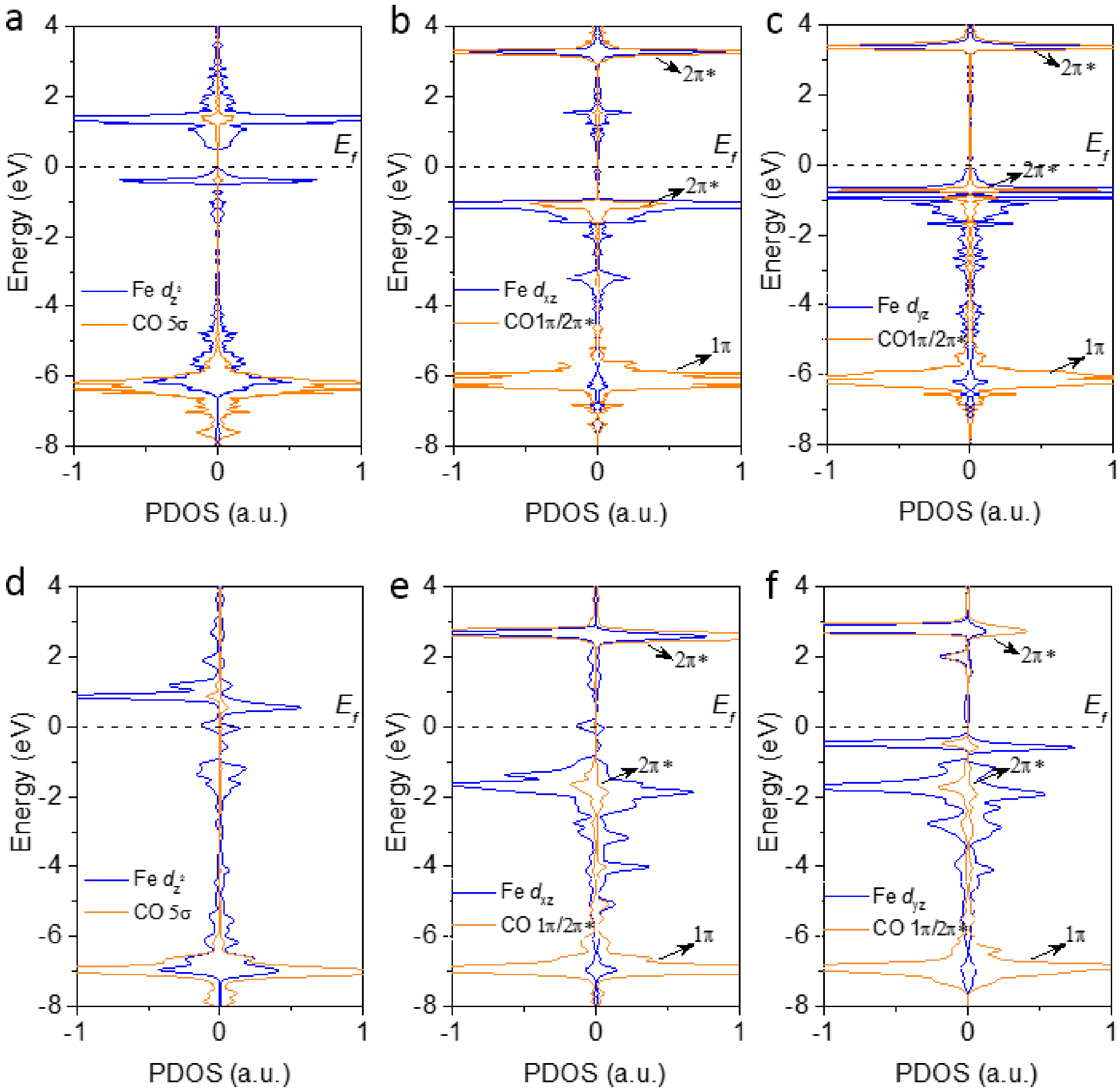

Supplementary Figure 29. Projected density of states of (a) Fe d_{z2}, CO 5σ, (b) Fe d_{xz}, CO 2π*, (c) Fe d_{yz}, CO 2π* in Fe-SAC-CO. Density of states of (d) Fe d_{z2}, CO 5σ, (e) Fe d_{xz}, CO 2π*, (f) Fe d_{yz}, CO 2π* in NiFe-DASC-CO.

Note: The CO frontier orbitals, 5σ and 2π*, are located closer to the Fermi level, making the 5σ and 2π* orbitals very close in energy to the metal *d*-band, which results the d_{z2}-5σ and d_{xz}/d_{yz}-2π* interactions[1,2].

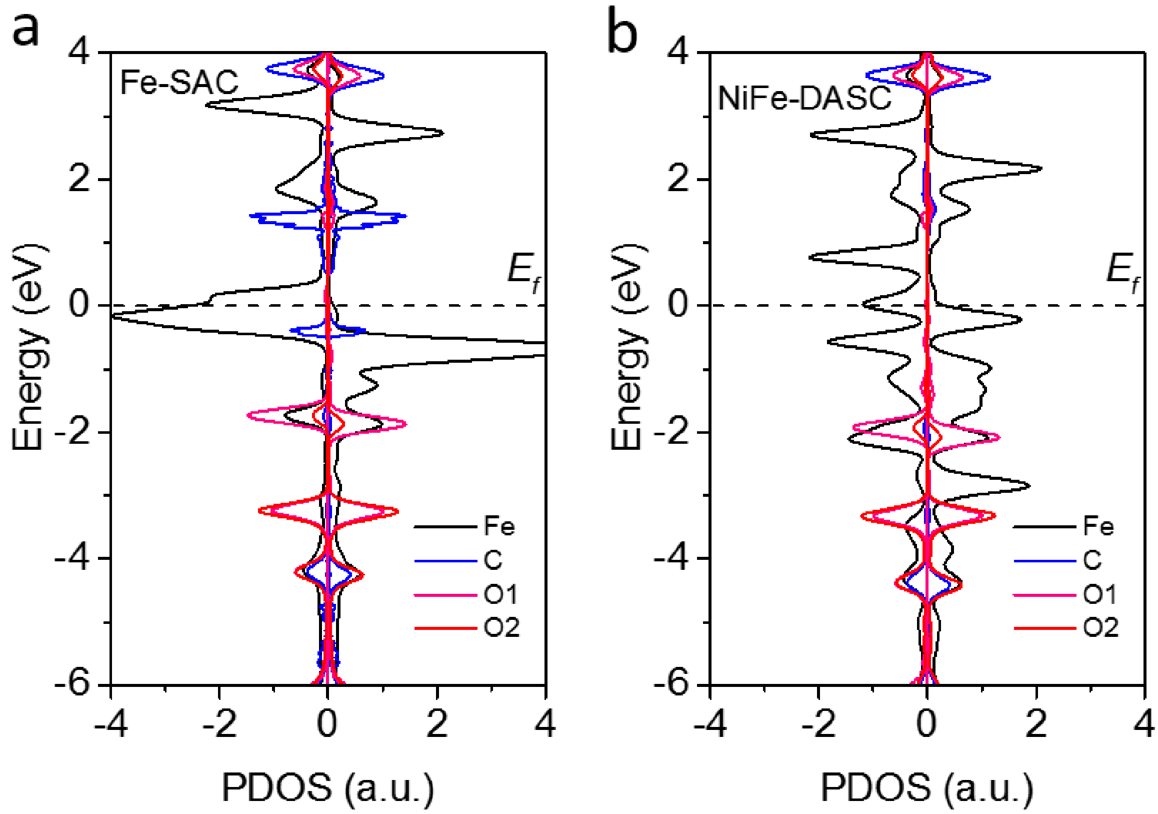

Supplementary Figure 30. Projected density of states (PDOS) of *COOH and Fe on (a) Fe-SAC and (b) NiFe-DASC.

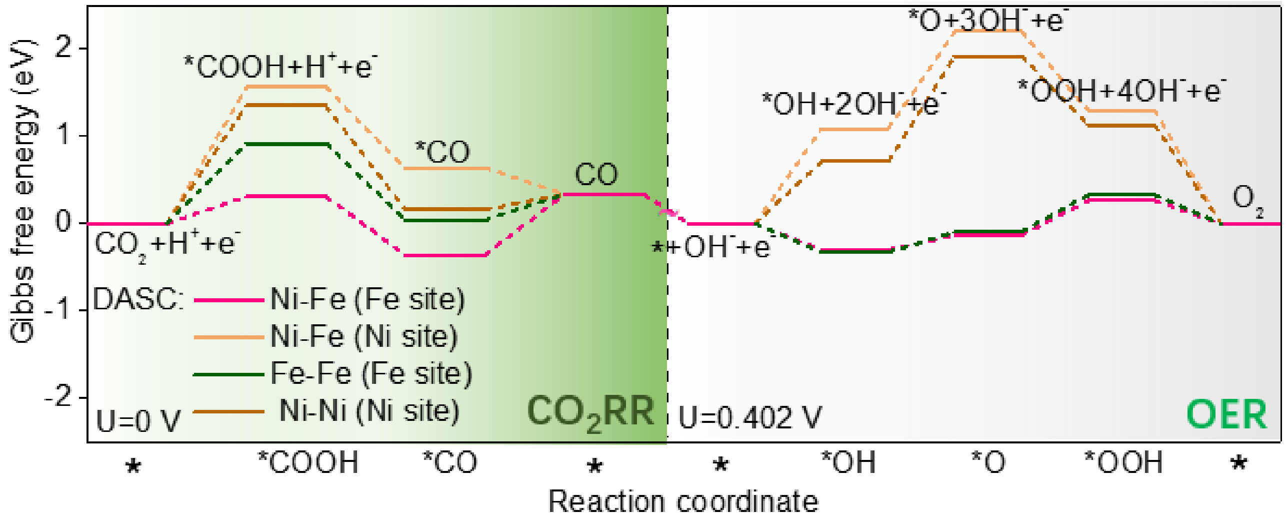

Supplementary Figure 31. Calculated free energy diagrams of DASCs for CO_2RR and OER processes.

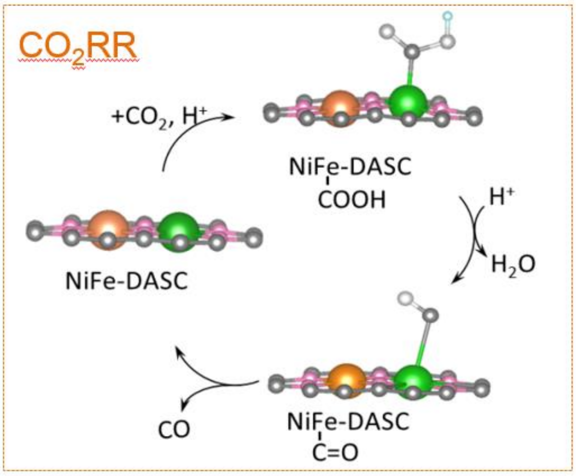

Supplementary Figure 32. The reaction pathways of NiFe-DASC for CO_2RR. Brown, green, pink, and grey balls are Ni, Fe, N, and C atoms, respectively.

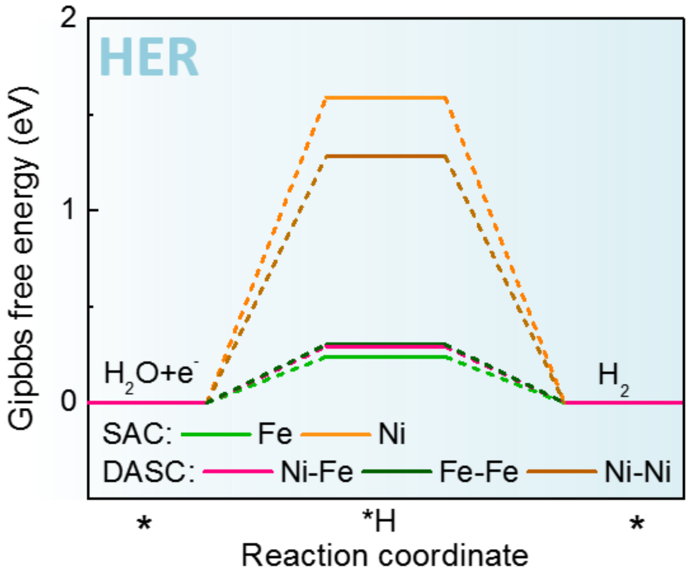

Supplementary Figure 33. The calculated free energy diagrams for HER processes.

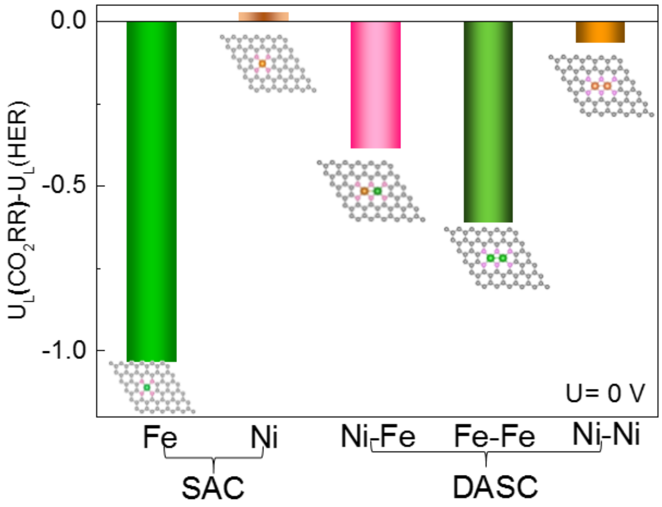

Supplementary Figure 34. Difference between the limiting potentials of CO_2RR and HER for different atomic catalysts.

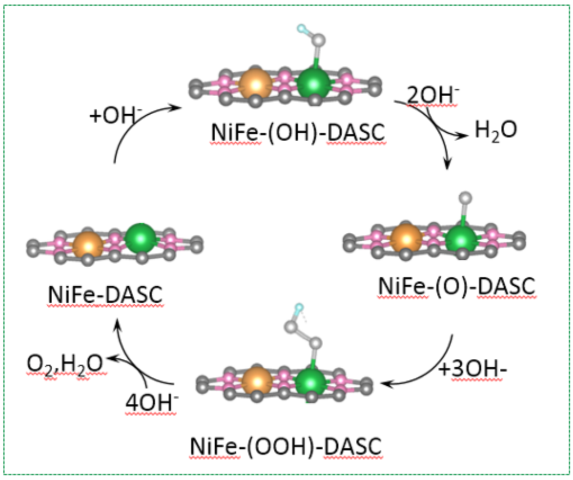

Supplementary Figure 35. The reaction pathways of NiFe-DASC for OER. Brown, green, pink, and grey balls are Ni, Fe, N, and C atoms, respectively.

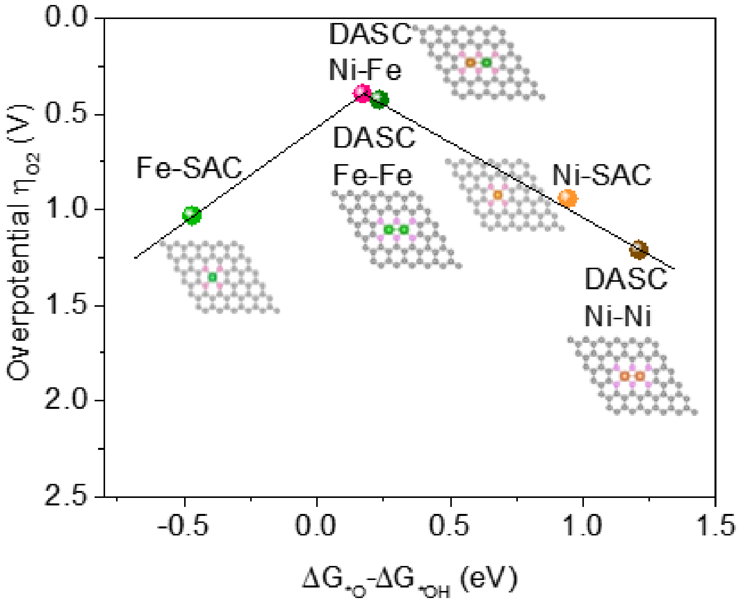

Supplementary Figure 36. Volcano plots of overpotential η of OER versus ΔG_{*O}-ΔG_{*OH}.

Supplementary Table 1. Elemental composition for the catalysts.

Sample	Nickel (wt.%)	Iron (wt.%)
Ni-SAC	3.48	/
Fe-SAC	/	3.34
NiFe-DASC	4.05	3.24

Supplementary Table 2. Comparison of the CO_2RR performance of NiFe-DASC with other state-of-the-art catalysts.

Catalysts	Electrolyte	Onset potential (vs. RHE, V)	Over-potential (mV)	Current density (mA cm^{-2})	Products	Faradaic efficiency (CO) (%)	Stability	Ref.
NiFe-DASC	0.5 M $KHCO_3$	-0.21	690	50.4	CO, H_2	94.5	>30 h	This work
Ni/Fe-N-C	0.5 M $KHCO_3$	-	880	19.7	CO, H_2	98	30 h	3
Ni-N-MEGO	0.5 M $KHCO_3$	-0.29	580	26.8	CO, H_2	92.1	24 h	4
NiPor-CTF	0.5 M $KHCO_3$	-0.44	490	7.0	CO, H_2	91	20h	5
A-Ni-NSG	0.5 M $KHCO_3$	-0.18	610	23.5	CO	94	100h	6
Ni SAC-NG	0.5 M $KHCO_3$	-	620	11	CO, H_2	95	8h	7
C-Zn_1Ni_4	0.5 M $KHCO_3$	-	920	71.5	CO, H_2	98	2h	8
Ni-N_x-C	0.1 M $KHCO_3$	-	550	9.5	CO, H_2	85	-	9
Ni SAs/N-C	0.5 M $KHCO_3$	-0.57	880	7.37	CO	71.9	60h	10
NiN-GS	0.1 M $KHCO_3$	-0.35	700	4.0	CO, H_2	93.2	20h	11
Ni_2-CPD_{Py}	0.1 M $KHCO_3$	-	780	0.55	CO, H_2	87	-	12
Cu/Ni(O	0.5M	-0.37	390	4.3	CO, H_2	92	22h	13

H)$_2$	$NaHCO_3$							
FePGH	0.1 M $KHCO_3$	-	280	~4.1	CO	95	20h	14
Fe/NG	0.1 M $KHCO_3$	-	480	~7.2	CO CH_4 H_2	80	10h	15
Fe-N_4	0.1 M $KHCO_3$	-	470	~3.2	CO, H_2	93	20h	16
Fe-N-C	0.1 M KHCO3	-0.3	480	7.5	CO, H_2	91	6h	17
CoPc©Fe-N-C	0.5 M KOH	-0.13	440	77.8	CO, H_2	90	20h	18

Supplementary Table 3. Comparison of oxygen evolution performance of the reported catalysts.

**Catalysts**	**Onset overpotential (mV vs. RHE)**	**η_{10} overpotential (mV vs. RHE)**	**Electrolyte**	**Ref.**
NiFe-DASC	250	310	1 M KOH	This work
Fe-NiNC-50	270	340	1 M KOH	19
Co_2Fe_1@NC	-	420	0.1 M KOH	20
UNT Co SAs/N-C	290	380	0.1 M KOH	21
CoXNi-N/C	310	360	0.1 M KOH	22
NiFe@g-C_3N_4/CNT	-	326	1 M KOH	23
HG-NiFe	-	310	1 M KOH	24
CoSAs@CNTs	300	410	0.1 M KOH	25
P/CoS_2@TiO_2 NPFs	180	260	0.1 M KOH	26
C-MOFC2-900	270	350	0.1 M KOH	27
Mo-N/C@MoS2	-	390	0.1 M KOH	28
S,N-Fe/N/C-CNT	-	370	0.1 M KOH	29
Co-C_3N_4/CNT	270	380	0.1 M KOH	30
Co/N-CNTs	-	390	0.1 M KOH	31
N-GRW	300	360	1 M KOH	32
Fe_2/Co_1-GNCL	-	350	1 M KOH	33

Supplementary Table 4. The discharge and charge performance of Zn-CO_2 batteries.

Cathode	Discharge voltage (V)	Charge voltage (V)	Faradaic efficiency (CO) (%)	Stability	Ref.
NiFe-DASC	0.19(5 mA cm^{-2}) 0.89(1 mA cm^{-2})	2.52(2.5 mA cm^{-2}) 2.36 (1 mA cm^{-2})	90(5 mA cm^{-2})	90h	This work
Ir@Au[34]	0.69(0.56 mA cm^{-2})	2.25(0.056 mA cm^{-2})	90(1.5 mA cm^{-2})	30h	Adv. Mater. 2019, 31, 1807807
Cu–N_2/GN[35]	0.7(1.0 mA cm^{-2})	2.4 (1.0 mA cm^{-2})	64(1.4 mA cm^{-2})	40h	Adv. Funct. Mater. 2019, 1907658
SiNC[36]	0.47 (0.125 mA cm^{-2})	-	85 (0.71 mA cm^{-2})	6h	Angew. Chem. 2018, 57, 13135
NiPG[37]	0.47(0.25 mA cm^{-2})	2.58(0.25 mA cm^{-2})	66(0.725 mA cm^{-2})	13h	J. Mater. Chem. A 2019, 7, 2575
Pd interconnected nanosheets[38]	0.78(0.56 mA cm^{-2})	0.96(0.56 mA cm^{-2})	81.2(0.56 mA cm^{-2}, for HCOOH)	33h	Angew. Chem. 2018, 130, 17242

Supplementary Table 5. Bader charge of metal atoms in samples.

Configuration		Bader charge (e)
NiFe-DASC	Ni	+0.84
	Fe	+1.08
Ni-SAC	Ni	+0.85
Fe-SAC	Fe	+1.06

Supplementary Table 6. Gibbs free energy change (ΔG) of the formation of different intermediates in CO_2RR and HER (U=0V).

	Intermediates	Ni-SAC (eV)	Fe-SAC (eV)	NiFe-DASC (Fe site) (eV)	NiNi-DASC (Ni site) (eV)	FeFe-DASC (Fe site) (eV)
CO_2RR	*COOH	1.56	0.33	0.31	1.35	0.92
	*CO	-1.36	-1.27	-0.66	-1.19	-0.88
	CO	0.13	1.28	0.69	0.18	0.30
HER	*H	1.59	0.24	0.29	1.283	0.304
$U_L(CO_2RR) - U_L(HER)$	/	0.03	-1.04	-0.4	-0.067	-0.616

Supplementary Table 7. Calculated zero-point energy (ZPE) and entropy (S) of gas- or liquid-phase molecules and adsorbates on the three modeled systems at T= 298 K.

	Species	ZPE (eV)	TS (eV)
Free molecules	H_2 (g)	0.28	0.40
	H_2O (l)	0.58	0.67
	CO_2	0.31	0.66
	CO	0.17	0.61
NiFe-DASC	*COOH	0.61	0.15
	*CO	0.22	0.09
	*OH	0.35	0.08
	*O	0.07	0.05
	*OOH	0.45	0.13
Fe-SAC	*COOH	0.63	0.14
	*CO	0.23	0.09
	*OH	0.35	0.08
	*O	0.07	0.05
	*OOH	0.44	0.13
Ni-SAC	*COOH	0.61	0.16
	*CO	0.23	0.09
	*OH	0.34	0.09
	*O	0.04	0.07
	*OOH	0.42	0.17

Supplementary Table 8. Formation energy of intermediates adsorbed on different sites of NiFe-DASC.

Adsorption of intermediates	Formation energy (eV)
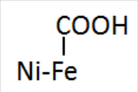	0.31 (for CO_2RR)
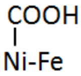	1.56
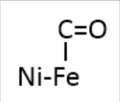	-0.36 (for CO_2RR)
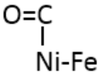	0.64
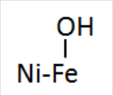	-0.30 (for OER)
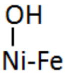	1.09
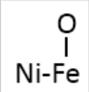	-0.13 (for OER)
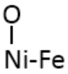	2.20
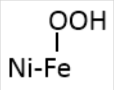	0.26 (for OER)
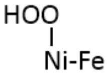	1.30

References

1. Schimka, L. et al. Accurate surface and adsorption energies from many-body perturbation theory. *Nat. Mater.* **9**, 741-744 (2010).
2. Gameel, K. M. et al. Unveiling CO adsorption on Cu surfaces: new insights from molecular orbital principles. *Phys. Chem. Chem. Phys.* **20**, 25892-25900 (2018).
3. Ren, W. et al. Isolated diatomic Ni-Fe metal-nitrogen sites for synergistic electroreduction of CO2. *Angew. Chem. Int. Ed.* **58**, 6972-6976 (2019).
4. Cheng, Y. et al. Unsaturated edge-anchored Ni single atoms on porous microwave exfoliated graphene oxide for electrochemical CO2. *Appl. Catal. B: Environ.* **243**, 294-303 (2019).
5. Lu, C. et al. Atomic Ni Anchored Covalent Triazine Framework as High Efficient Electrocatalyst for Carbon Dioxide Conversion. *Adv. Funct. Mater.* **29**, 1806884 (2019).
6. Yang, H. B. et al. Atomically dispersed Ni(i) as the active site for electrochemical CO2 reduction. *Nat. Energy* **3**, 140-147 (2018).
7. Jiang, K. et al. Isolated Ni single atoms in graphene nanosheets for high-performance CO2 reduction. *Energy & Environ. Sci.* **11**, 893-903 (2018).
8. Yan, C. et al. Coordinatively unsaturated nickel-nitrogen sites towards selective and high-rate CO2 electroreduction. *Energy & Environ. Sci.* **11**, 1204-1210 (2018).
9. Ju, W. et al. Understanding activity and selectivity of metal-nitrogen-doped carbon catalysts for electrochemical reduction of CO2. *Nat. Commun.* **8**, 944 (2017).
10. Zhao, C. et al. Ionic Exchange of Metal-Organic Frameworks to Access Single Nickel Sites for Efficient Electroreduction of CO2. *J. Am. Chem. Soc.* **139**, 8078-8081 (2017).
11. Jiang, K. et al. Transition-metal single atoms in a graphene shell as active centers for highly efficient artificial photosynthesis. *Chem* **3**, 950-960 (2017).
12. Nishihara, H. et al. Synthesis of ordered carbonaceous frameworks from organic crystals. *Nat. Commun.* **8**, 109 (2017).
13. Dai, L. et al. Ultrastable atomic copper nanosheets for selective electrochemical reduction of carbon dioxide. *Sci. Adv.* **3**, e1701069 (2017).
14. Choi, J. et al. Energy efficient electrochemical reduction of CO2 to CO using a three-dimensional porphyrin/graphene hydrogel. *Energy & Environ. Sci.* **12**, 747-755 (2019).
15. Zhang, C. et al. Electrochemical CO2 reduction with atomic iron-dispersed on nitrogen-doped graphene. *Adv. Energy Mater.* **8**, 1703487 (2018).
16. Pan, F. et al. Unveiling active sites of CO2 reduction on nitrogen-coordinated and atomically dispersed iron and cobalt catalysts. *ACS Catal.* **8**, 3116-3122 (2018).
17. Huan, T. N. et al. Electrochemical reduction of CO2 catalyzed by Fe-N-C materials: A structure-selectivity study. *ACS Catal.* **7**, 1520-1525 (2017).
18. Long, L. et al. Synergistic catalysis over iron-nitrogen sites anchored with cobalt phthalocyanine for efficient CO2 electroreduction. *Adv. Mater.* **31**, 1903470 (2019).
19. Zhu, X. et al. Harnessing the interplay of Fe-Ni atom pairs embedded in nitrogen-doped carbon for bifunctional oxygen electrocatalysis. *Nano Energy* **71**, 104597 (2020).
20. Tang, T. et al. Metastable rock salt oxide-mediated synthesis of high-density dual-protected M@NC for long-life rechargeable zinc-air batteries with record power density. *J. Am. Chem. Soc.*, **142**, 7116-7127 (2020).
21. Sun, X. et al. High-performance single atom bifunctional oxygen catalysts derived from ZIF-67 superstructures. *Nano Energy* **61**, 245-250 (2019).
22. Li, Z. et al. Atomic Co/Ni dual sites and Co/Ni alloy nanoparticles in N-doped porous Janus-like carbon

frameworks for bifunctional oxygen electrocatalysis. *Appl. Catal. B: Environ.* **240**, 112-121 (2019).
23. Wu, C. et al. Insight into the role of Ni-Fe dual sites in the oxygen evolution reaction based on atomically metal-doped polymeric carbon nitride. *J. Mater. Chem. A* **7**, 14001-14010 (2019).
24. Wang, J. et al. In situ formation of molecular Ni-Fe active sites on heteroatom-doped graphene as a heterogeneous electrocatalyst toward oxygen evolution. *Sci. Adv.* **4**, eaap7970 (2018).
25. Dilpazir, S. et al. Cobalt single atoms immobilized N-doped carbon nanotubes for enhanced bifunctional catalysis toward oxygen reduction and oxygen evolution reactions. *ACS Appl. Energy Mater.* **1**, 3283-3291 (2018).
26. Guo, L. et al. N, P-doped CoS2 embedded in TiO2 nanoporous films for Zn-air batteries. *Adv. Funct. Mater.* **28**, 1804540 (2018).
27. Zhang, M. et al. Novel MOF-derived Co@N-C bifunctional catalysts for highly efficient Zn-air batteries and water splitting. *Adv. Mater.* **30**, 1705431 (2018).
28. Amiinu, I. S. et al. Multifunctional Mo-N/C@MoS2 electrocatalysts for HER, OER, ORR, and Zn-air batteries. *Adv. Funct. Mater.* **27**, 1702300 (2017).
29. Chen, P. et al. Atomically dispersed iron-nitrogen species as electrocatalysts for bifunctional oxygen evolution and reduction reactions. *Angew. Chem. Int. Ed.* **56**, 610-614 (2017).
30. Zheng, Y. et al. Molecule-level g-C3N4 coordinated transition metals as a new class of electrocatalysts for oxygen electrode reactions. *J. Am. Chem. Soc.* **139**, 3336-3339 (2017).
31. Liu, Y. et al. Transition metals (Fe, Co, and Ni) encapsulated in nitrogen-doped carbon nanotubes as bi-functional catalysts for oxygen electrode reactions. *J. Mater. Chem. A* **4**, 1694-1701 (2016).
32. Yang, H. B. et al. Identification of catalytic sites for oxygen reduction and oxygen evolution in N-doped graphene materials: Development of highly efficient metal-free bifunctional electrocatalyst. *Sci. Adv.* **2**, e1501122 (2016).
33. Wang, Y. S. et al. Fabricating dual-atom iron catalysts for efficient oxygen evolution reaction: A heteroatom modulator approach. *Angew. Chem. Int. Ed.* **132**, 16147 (2020)
34. Wang, X. et al. Rechargeable Zn-CO2 electrochemical cells mimicking two-step photosynthesis. *Adv. Mater.* **31**, 1807807 (2019).
35. Zheng, W. et al. Atomically defined undercoordinated active sites for highly efficient CO2 electroreduction. *Adv. Funct. Mater.* **30**, 1907658 (2020).
36. Ghausi, M. A. et al. CO2 overall splitting by a bifunctional metal-free electrocatalyst. *Angew. Chem. Int. Ed.* **57**, 13135-13139 (2018).
37. Yang, R. et al. A trifunctional Ni-N/P-O-codoped graphene electrocatalyst enables dual-model rechargeable Zn-CO2/Zn-O2 batteries. *J. Mater. Chem. A* **7**, 2575-2580 (2019).
38. Xie, J. et al. Reversible aqueous zinc-CO2 batteries based on CO2-HCOOH interconversion. *Angew. Chem. Int. Ed.* **57**, 16996-17001 (2018).
